# Multi-omics integration reveals that pyrimidine metabolism in lung adenocarcinoma drives an immunosuppressive microenvironment

**DOI:** 10.1016/j.isci.2026.115326

**Published:** 2026-03-18

**Authors:** Miaoyan Liu, Houqiang Li, Shenghan Xu, Yike Zhou, Min Yao, Jiahai Shi, Lou Zhong

**Affiliations:** 1 Department of Thoracic Surgery, Affiliated Hospital of Nantong University, Nantong, China; 2 Department of Immunology, Medical School of Nantong University and Research Center of Clinical Medicine, Affiliated Hospital of Nantong University, Nantong, China

**Keywords:** Molecular biology, Immunology, Bioinformatics

## Abstract

Metabolic reprogramming in lung adenocarcinoma (LUAD) profoundly shapes the tumor immune microenvironment (TIME), yet the immune-regulatory role of pyrimidine metabolism remains unclear. This study investigates the mechanisms and clinical significance of pyrimidine metabolism in LUAD. Multi-omics analysis identified pyrimidine metabolism as a key prognostic pathway. A machine learning-based risk model revealed nine core genes, with *MCM7* as a central driver. Single-cell and spatial transcriptomic analyses showed that high-pyrimidine-metabolism tumor cells interact with immune cells via enhanced migration inhibitory factor (MIF) signaling. Functional assays confirmed that *MCM7* knockdown suppresses LUAD proliferation and migration. Mechanistically, MCM7 regulates pyrimidine synthesis enzymes (DHODH and UMPS), activates the ERK pathway, and interacts with the MIF-CD74 axis. These findings indicate that *MCM7* serves as a critical link connecting pyrimidine metabolic reprogramming to the regulation of the TIME in LUAD.

## Highlights

• MCM7 links pyrimidine metabolism to immunosuppression
• High MCM7 expression predicts poor LUAD prognosis
• MCM7 regulates DHODH/UMPS and activates ERK signaling
• MCM7 interacts with the MIF-CD74 immune axis

## Introduction

Lung adenocarcinoma (LUAD), the predominant subtype of non-small cell lung cancer (NSCLC), remains a leading cause of cancer-related morbidity and mortality. Although targeted and immune-based therapies have markedly improved patient management, clinical responses remain heterogeneous, and a substantial proportion of individuals exhibit primary or acquired resistance. Emerging evidence underscores the pivotal contributions of the tumor microenvironment (TME) and metabolic reprogramming to LUAD progression.^1^^,^^2^^,^^3^^,^^4^

Dysregulated metabolism is a cardinal hallmark of cancer. Pyrimidine biosynthesis, the quintessential pathway for nucleotide supply, fuels neoplastic proliferation and, concomitantly, sculpts an immunosuppressive TME.^5^^,^^6^^,^^7^^,^^8^ Cancer cells exploit urea cycle defects to overproduce pyrimidines, thereby enhancing mutational load (R→Y transitions) and neo-antigenicity while simultaneously releasing ATP during stress or death.^9^ Extracellular ATP, if not promptly catabolized by CD39/CD73, engages P2RY2/P2RX7 receptors to activate dendritic cells and other immune subsets, tipping the balance toward immunostimulation.^10^^,^^11^ Conversely, high CD39/CD73 activity converts ATP to adenosine, fostering immune tolerance. Pyrimidine metabolism further molds an immunosuppressive niche by promoting M2 macrophage polarization, CD8^+^ T cell exhaustion, and upregulation of immune checkpoints.^12^^,^^13^^,^^14^ Aberrant expression of pyrimidine enzymes such as dihydroorotate dehydrogenase (*DHODH*) and uridine monophosphate synthetase (*UMPS*) correlates with tumor progression and chemoresistance across malignancies^15^^,^^16^^,^^17^^,^^18^; yet, their precise roles in LUAD—particularly how they rewire the TME via intercellular crosstalk—remain elusive.

Within the tumor immune microenvironment, T cell exhaustion constitutes a pivotal determinant of immunotherapy failure.^19^ It is characterized by progressive loss of CD8^+^ T cell effector functions—evidenced by diminished IL-2, TNF-α, and IFN-γ secretion—and sustained upregulation of inhibitory receptors including PD-1 and Tim-3.^20^ Tumor-derived metabolites such as lactic acid and kynurenine can directly impair T cell activity.^21^^,^^22^ Notably, macrophage migration inhibitory factor (*MIF*) functions as a central hub linking inflammation and metabolism, orchestrating tumor-immune cell crosstalk.^23^^,^^24^ Via the CD74-NF-κB axis, MIF reprograms mitochondrial dynamics and energy metabolism in malignant cells, fostering tumor survival and proliferation.^25^ Nevertheless, how pyrimidine metabolism governs T cell exhaustion through MIF-dependent pathways remains undefined and warrants further investigation.

Integrating multi-omics data, we demonstrate, that minichromosome maintenance complex component 7 (*MCM7*) accelerates LUAD progression by remodeling pyrimidine metabolism and potentiating the MIF signaling axis. Transcriptomic profiling and single-cell RNA sequencing revealed that malignant LUAD cells with heightened pyrimidine flux establish amplified MIF-mediated crosstalk with CD8^+^ T cells and macrophages, concomitant with accentuated T cell exhaustion. Functional assays further establish *MCM7* as a master regulator whose expression tightly correlates with MIF pathway activity and an immunosuppressive phenotype. These findings delineate a metabolic-immune circuitry in LUAD and provide a mechanistic rationale for therapeutic targeting of the *MCM7-*pyrimidine metabolism-MIF axis.

## Results

### Metabolic pathway scoring and key pathway screening in LUAD patients

**Figure 1:**
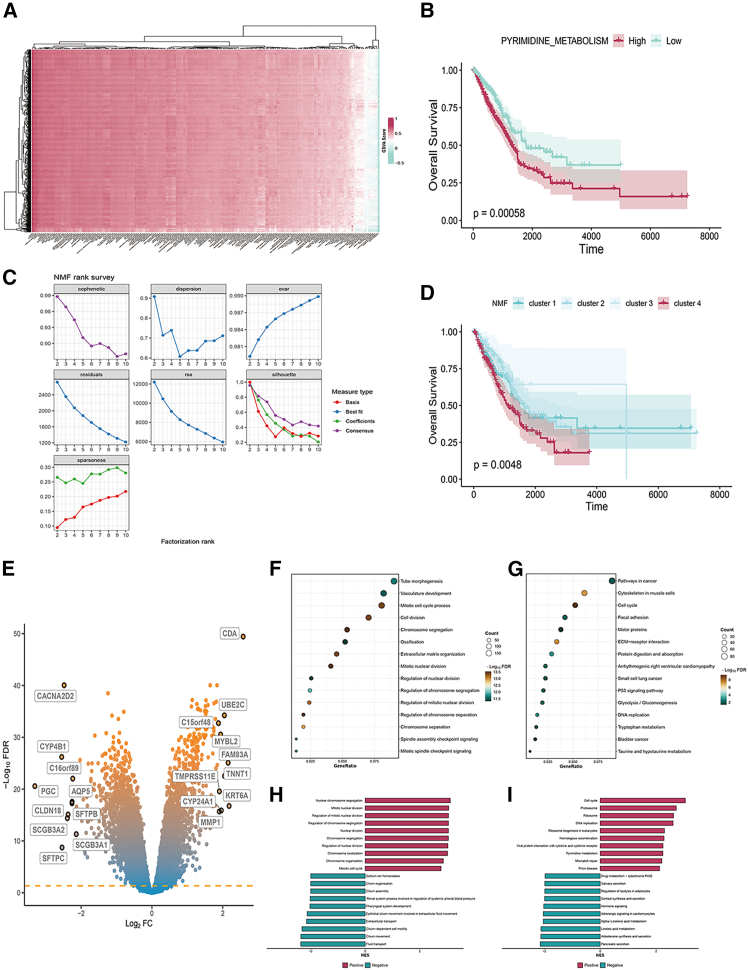
Metabolic pathway scoring and key pathway screening in LUAD patients (A) Heatmap of KEGG pathway GSVA scores in LUAD patients. (B) K-M survival curve of LUAD patients’ pyrimidine metabolism scores and patient survival. (C) Rank curve of NMF-related parameters. (D) K-M survival curves of LUAD patients in different NMF clusters. (E) Volcano plot of DEGs between LUAD cluster 4 patients and other patients. (F) GO enrichment analysis of DEGs. (G) KEGG enrichment analysis of DEGs. (H) GSEA-GO enrichment analysis of DEGs. (I) GSEA-KEGG enrichment analysis of DEGs.

GSVA of KEGG metabolic pathways in LUAD revealed heterogeneous pathway activities across patients (Figure 1A). We therefore correlated each metabolic score with overall survival (OS) and retained pathways with *p* < 0.001; Kaplan-Meier (K-M) analysis confirmed six pathways as prognostic (Figure S1). Among these, pyrimidine metabolism was selected for focused investigation^26^; patients with high pyrimidine scores exhibited significantly worse OS (Figure 1B). Using non-negative matrix factorization (NMF) on pyrimidine metabolism-related genes, we identified four robust clusters (Figures 1C and S2). Cluster 4 displayed the poorest prognosis (Figure 1D). Differential expression between cluster 4 and the remaining cohort revealed 1,247 genes (Figure 1E). Functional enrichment (GO, KEGG, and GSEA) showed significant over-representation of nucleic acid and metabolic pathways (Figures 1F–1I).

### Single-cell analysis and pyrimidine metabolism scoring in LUAD patients

**Figure 2:**
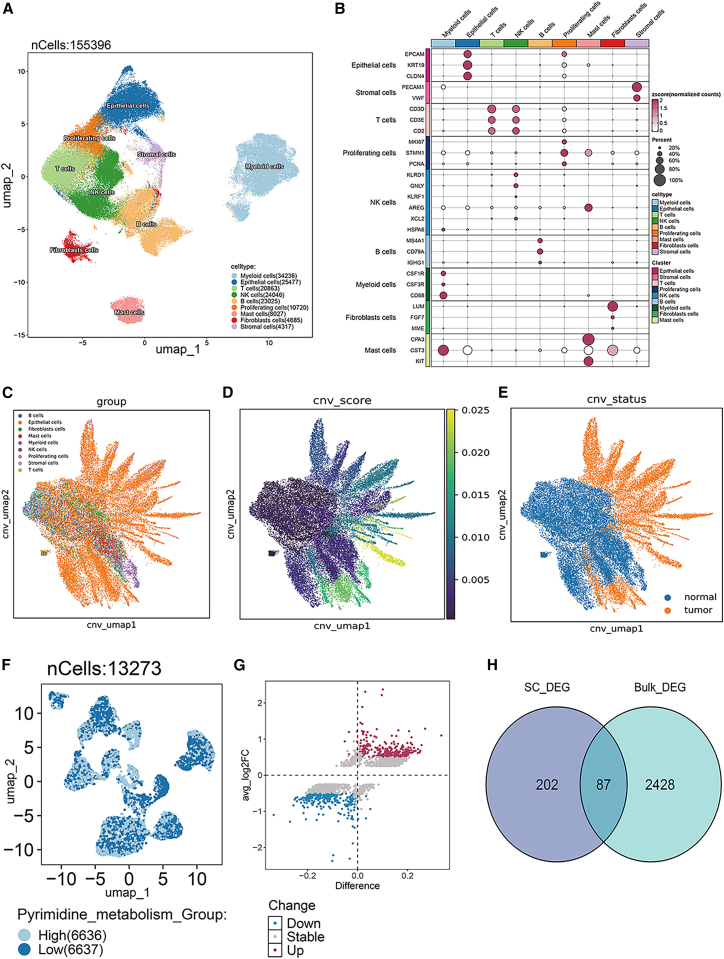
Single-cell analysis and pyrimidine metabolism scoring in LUAD patients (A) UMAP clustering diagram of single-cell data from LUAD patients. (B) Dotplot diagram of single-cell subpopulations in LUAD patients. (C) UMAP clustering diagram of CNV scores for single-cell subpopulations. (D) CNV scoring plot of single-cell subpopulations. (E) Malignant cell identification results of single-cell subpopulations. (F) UMAP clustering plot of malignant cells grouped by pyrimidine metabolism scoring. (G) Volcano plot of DEGs between high and low pyrimidine metabolism groups in malignant cells. (H) Venn diagram of DEGs in single cells and the general transcriptome.

Single-cell RNA-seq profiles from LUAD patients were integrated, yielding 155,396 high-quality cells resolved into nine transcriptionally distinct subtypes (Figure 2A). Canonical marker-gene expression across clusters is summarized in the dot plot (Figure 2B). Malignant epithelial cells were identified with inferCNV based on elevated copy number variation (CNV) scores that deviated markedly from non-malignant cells (Figures 2C, 2D, and S3). In total, 13,273 malignant cells were retained for downstream analyses (Figure 2E). Malignant cells were stratified into PyrMetHigh and PyrMetLow subsets using scMetabolism pyrimidine-metabolism scores (Figures 2F and S4). Despite comparable global transcriptional landscapes (UMAP overlay), 1,232 differentially expressed genes (DEGs) were identified between the groups (Figure 2G and Table S1). Intersection of these DEGs with bulk-derived prognostic signatures yielded 87 high-confidence candidate markers (Figure 2H).

Table S1. Differentially expressed genes between PyrMet_High and PyrMet_Low subsets in malignant cells, related to Figure 2

### Marker gene screening and risk scoring model construction

**Figure 3:**
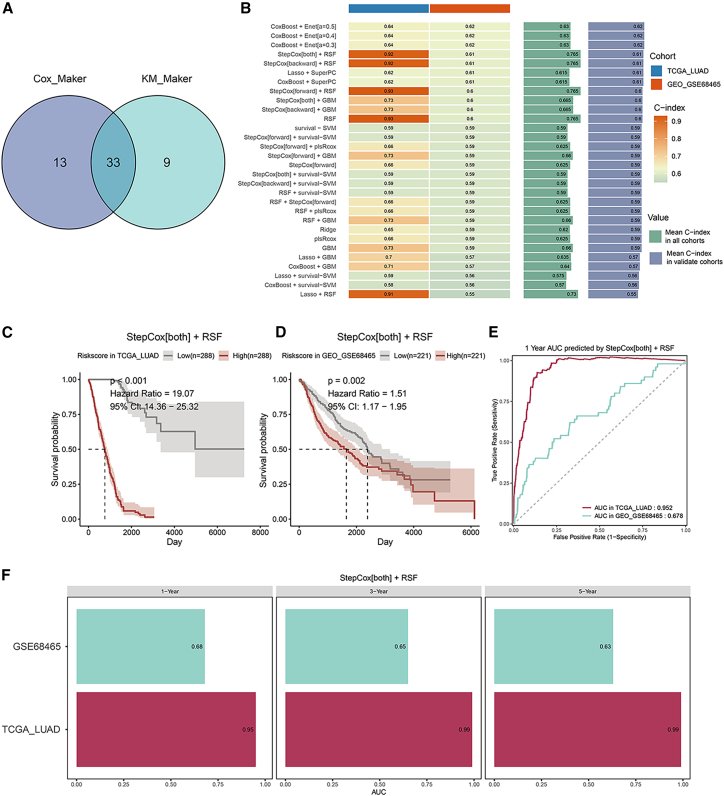
Marker gene screening and risk scoring model construction (A) Venn diagram of Cox analysis and K-M survival analysis of candidate marker genes. (B) Mime1 package for constructing risk prediction models, showing the top 30 models by C-index. (C) Survival K-M curves of the risk model in the training set. (D) Survival K-M curves of the risk model in the test set. (E) ROC curves for one-year survival in the training and test sets. (F) AUC values for 1-, 3-, and 5-year survival predictions in the training and test sets. (G) Forest plot. (H) Nomogram. (I) Multivariate Cox regression forest plot. Data are represented as mean ± SD where applicable.

Univariable Cox and K-M analyses of the 87 candidate genes identified 46 and 42 significant genes, respectively; their intersection yielded 33 prognostic markers (Figure 3A). After applying the unicox.filter.for.candi = TRUE criterion, 28 genes (*DTYMK*, *PERP*, *PHB*, *RAN*, *PAICS*, *SLC2A1*, *SRM*, *NME1*, *TIMP1*, *CKS1B*, *SLC6A8*, *TOMM40*, *ADM*, *PAFAH1B3*, *MRPL12*, *MRPS12*, *DSC2*, *NUDT1*, *LYPD3*, *CLIC1*, *MCM7*, *TXN*, *KRT6A*, *RPL39L*, *CHCHD2*, *TMSB10*, *PFDN2*, and *MRPL11*) were retained for model development. We next trained 101 machine-learning algorithms; the top 30 ranked by the concordance index (C-index) are shown in Figure 3B. The StepCox[both] + RSF model was selected for further analysis. Survival curves for high- and low-risk groups were plotted in the training and testing datasets (Figures 3C and 3D), showing that the survival prognosis of the high-risk group was significantly worse in both the training and testing datasets. One-year survival prediction receiver operating characteristic (ROC) curves were plotted in the training and testing datasets (Figure 3E). The area under the curve (AUC) values for 1-, 3-, and 5-year survival predictions were calculated in the training and testing datasets (Figure 3F), indicating that the risk model can effectively predict survival outcomes in LUAD patients. The StepCox[both] + RSF model identified nine prognostic marker genes: *PAICS*, *SLC2A1*, *NME1*, *CKS1B*, *ADM*, *MRPL12*, *DSC2*, *MCM7*, and *KRT6A* (Figures 3G and 3H). To verify whether the risk score could serve as an independent prognostic factor for patients with lung adenocarcinoma, we performed a multivariate Cox proportional hazards regression analysis. The covariates included in the analysis were: risk score, patient age, gender, and clinical stage. The multivariate Cox analysis demonstrated that the risk score in the constructed model was an independent prognostic factor (Figure 3I).

### T cell state scoring and immune infiltration analysis in LUAD patients

**Figure 4:**
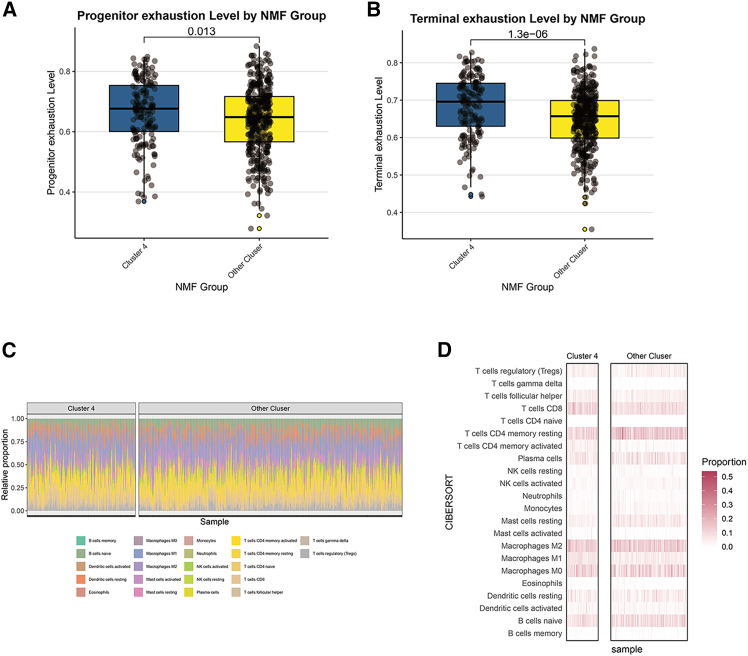
T cell state scoring and immune infiltration analysis in LUAD patients (A and B) Boxplot showing the difference in T cell initial exhaustion and terminal exhaustion state scores between LUAD cluster 4 patients and other patients. (C) Bar chart showing the difference in the proportion of different immune cell infiltration between cluster 4 patients and other patients. (D) Heatmap showing the infiltration of various types of immune cells in cluster 4 patients and other patients.

T cell state scoring was performed on LUAD patients, revealing that LUAD cluster 4 patients exhibited significantly elevated levels of initial and terminal exhaustion compared to other patients (Figures 4A and 4B). Immune infiltration analysis of LUAD patients revealed different immune cell infiltration proportions between cluster 4 patients and other patients (Figure 4C), as well as heat maps of various types of immune cell infiltration between cluster 4 patients and other patients (Figure 4D).

### LUAD cell communication analysis

**Figure 5:**
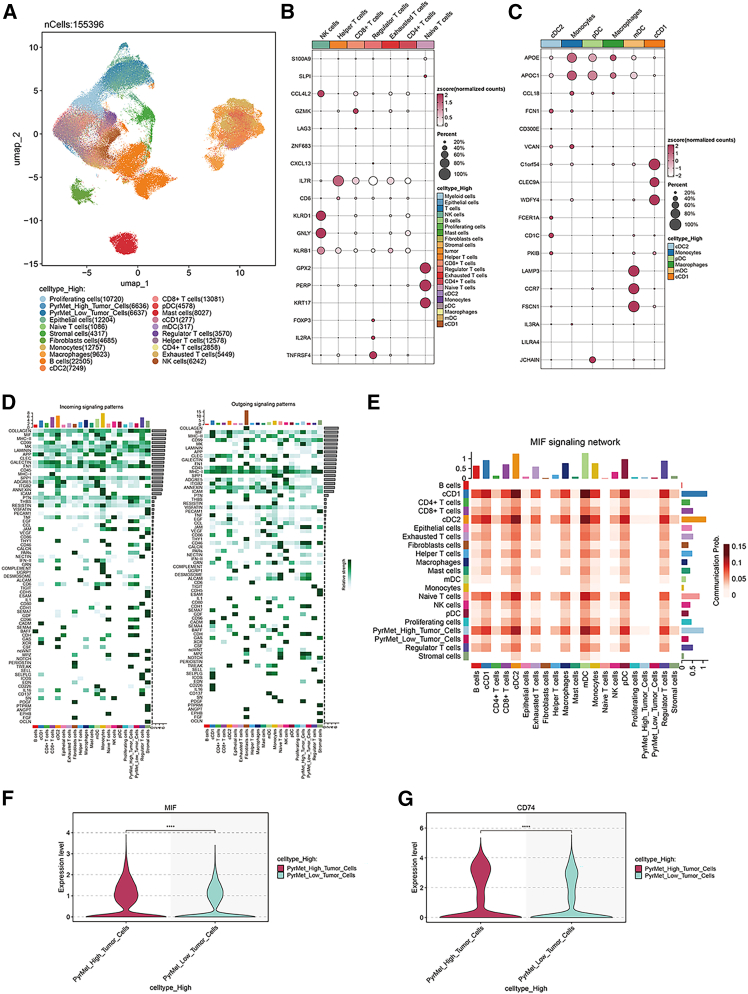
LUAD cell communication analysis (A) LUAD single-cell subpopulation UMAP clustering diagram. (B) T/NK cell subpopulation marker gene dot plot diagram. (C) Myeloid cell subpopulation marker gene dot plot diagram. (D) Heatmap of communication strength between cell subpopulations. (E) Heatmap of communication proportion of the MIF signaling pathway in each cell subpopulation. (F and G) Violin plots of differential expression of MIF and CD74 genes in the PyrMet_High and PyrMet_Low groups.

We further resolved the single-cell atlas into 21 distinct sub-populations of T/NK and myeloid cells (Figure 5A). Canonical marker genes for T/NK and myeloid subsets were visualized in dot plots (Figures 5B and 5C; Tables S2 and S3), and their corresponding UMAP embeddings are presented (Figures S5A and S5B). Cell-cell communication analysis revealed markedly divergent MIF signaling between the high-pyrimidine metabolism tumor cells (PyrMet_High) and low-pyrimidine metabolism tumor cells (PyrMet_Low) groups (Figure 5D). Heatmaps of MIF-mediated interactions showed that the PyrMet_High group engaged in significantly stronger signaling with CD8^+^ T cells, CD4^+^ T cells, and macrophages (Figure 5E), suggesting that this enhanced crosstalk accelerates LUAD progression. Consistently, violin plots demonstrated elevated expression of the MIF-CD74 axis components *MIF* and *CD74* in the PyrMet_High group (Figures 5F and 5G), corroborating the functional relevance of this pathway.

Table S2. Canonical marker genes for T/NK cell subpopulations, related to Figure 5

Table S3. Canonical marker genes for myeloid cell subpopulations, related to Figure 5

### Correlation between marker genes and immune cell infiltration and MIF expression

**Figure 6:**
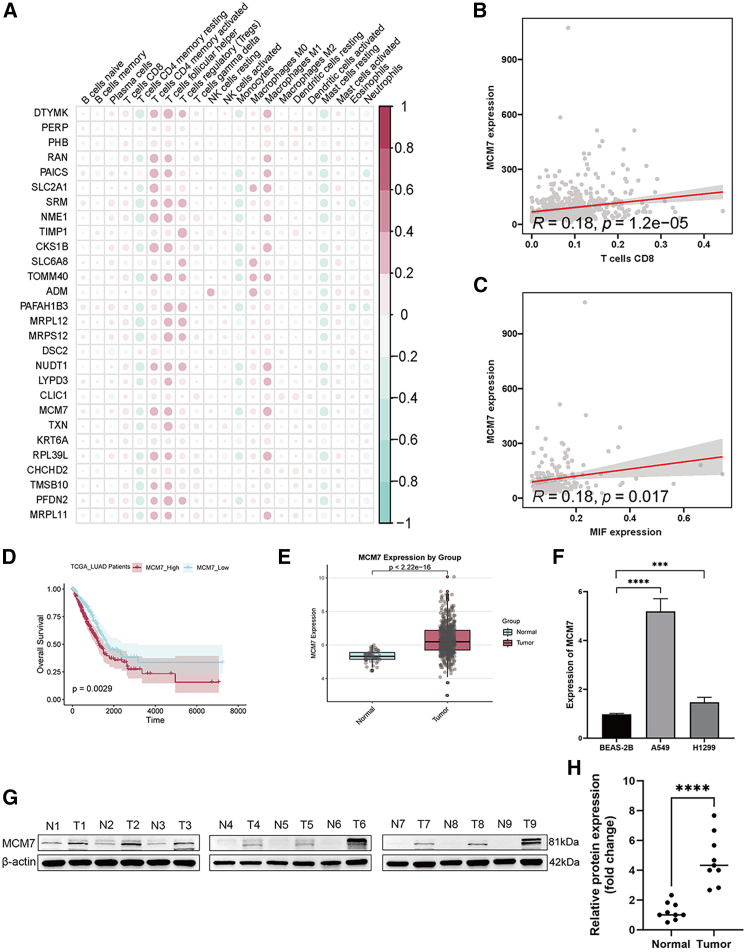
Correlation between marker genes and immune cell infiltration and MIF expression (A) Heatmap showing the correlation between marker genes and the degree of infiltration of various immune cells. (B) Regression plot showing the correlation between MCM7 and the degree of CD8^+^ T cell infiltration. (C) Regression plot showing the correlation between MCM7 and MIF expression levels. (D) OS K-M curves for patients with high- and low-expression of MCM7. (E) Boxplots of differential gene expression of MCM7 between LUAD patient samples and adjacent normal tissue samples. (F) qRT-PCR showed elevated MCM7 mRNA in A549/H1299 LUAD cells vs. HBE normal cells. (G and H) WB confirmed upregulated MCM7 protein in nine paired tumor vs. adjacent tissues. Data are represented as mean ± SD. Statistical significance was determined by two-tailed Student’s *t* test or log rank test. ns, not significant; ∗*p* < 0.05; ∗∗*p* < 0.01; ∗∗∗*p* < 0.001; ∗∗∗∗*p* < 0.0001.

Correlation analysis revealed that the identified marker genes exhibited positive correlations with CD8^+^ T cell and M2 macrophage infiltration (Figure 6A), with consistent expression patterns across markers. Among these, *MCM7* expression was positively correlated with CD8^+^ T cell abundance (r = 0.62, *p* < 0.001; Figure 6B) and *MIF* expression (r = 0.71, *p* < 0.001; Figure 6C). High *MCM7* expression predicted poor prognosis in LUAD patients (Figure 6D) and was significantly upregulated in tumor tissues compared to paired non-tumor adjacent tissues (NATs) (Figure 6E). *In vitro*, *MCM7* mRNA levels were elevated in LUAD cell lines (A549 and H1299) versus human bronchial epithelial cells (16HBE) (Figure 6F). Consistently, MCM7 protein expression was markedly increased in LUAD tissues relative to adjacent normal tissue (Figures 6G and 6H).

### CWGCNA

**Figure 7:**
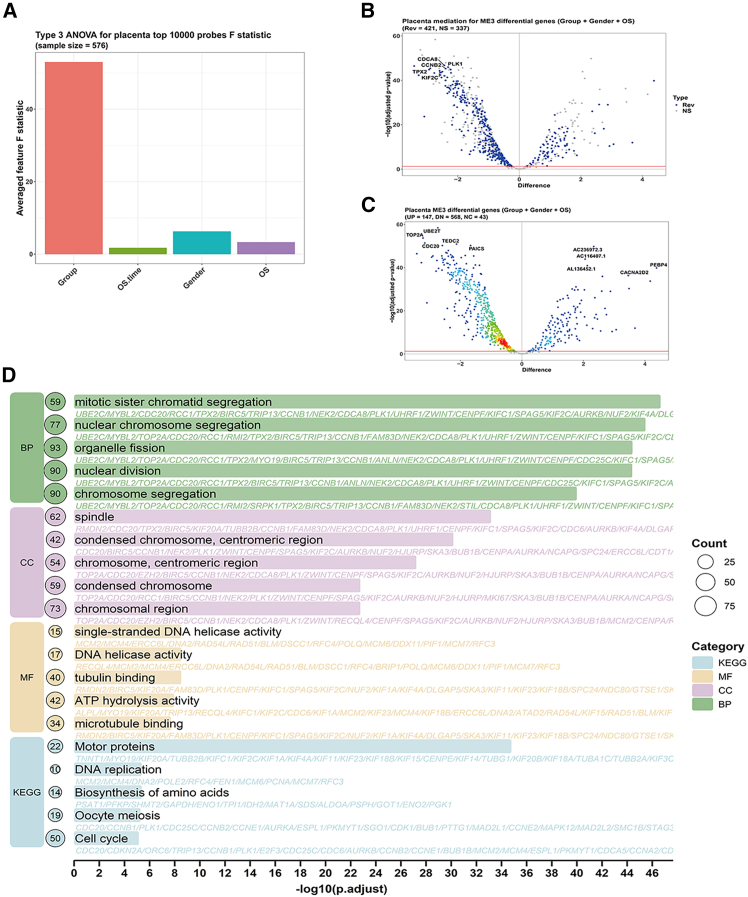
CWGCNA (A) Bar plot of variable variance. (B) Volcano plot of causal genes. (C) Volcano plot of upregulated/downregulated genes. (D) Functional enrichment analysis of ME3 genes.

In CWGCNA, four outcomes were incorporated: disease occurrence, gender, survival status, and survival time. Among these, disease occurrence explained the highest variance, while gender and survival status also exhibited substantial variance, indicating their roles as confounding factors (Figure 7A). Based on module analysis, we identified ME3, which showed 147 upregulated and 568 downregulated genes compared to controls. Among these, 421 genes mediated the causal pathway from disease occurrence → ME3 module → ME3 genes, with no evidence of forward causality. This suggests that LUAD development drives the expression or suppression of ME3-associated genes (Figures 7B and 7C). Functional enrichment analysis of ME3 genes revealed significant associations with pathways and biological processes including cell division, chromosomal segregation, and DNA helicase activity. Notably, the ME3 module contained *MCM7*—a gene previously identified via machine learning—along with its related family members (Figure 7D). We hypothesize that LUAD onset stimulates rapid cell proliferation, leading to upregulation of pyrimidine metabolism pathways. Thus, LUAD development acts as the cause, and pyrimidine metabolism serves as the effect.

### Virtual knockout and metabolic flux analysis

**Figure 8:**
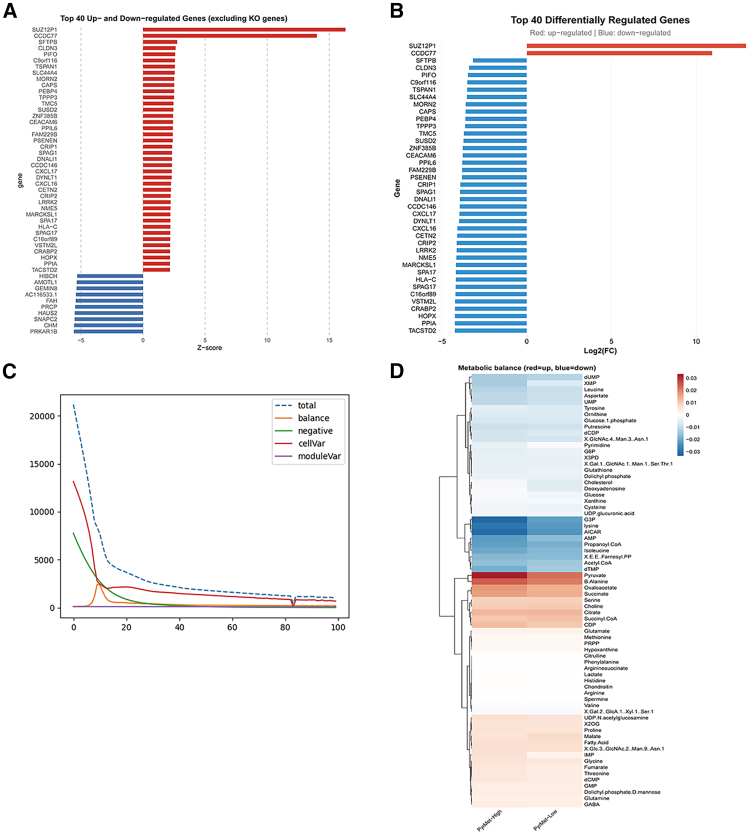
Virtual knockout and metabolic flux analysis (A) Volcano plot of *Z* scores post-knockout. (B) Volcano plot of LogFC values post-knockout. (C) Model fitting curves. (D) Metabolite heatmap.

Simulated knockout of *MCM7* revealed significant expression changes primarily in genes including *SUZ12P1*, *CCDC77*, and *SFTPB* (Figures 8A and 8B). Metabolic flux analysis demonstrated that in pyrimidine metabolism-high tumor cells, fluxes of pyruvate, alanine, oxaloacetate, and succinate were elevated, while fluxes of glycerol-3-phosphate (G3P), lysine, and adenosine analogs were reduced (Figures 8C and 8D).

### CellChat

**Figure 9:**
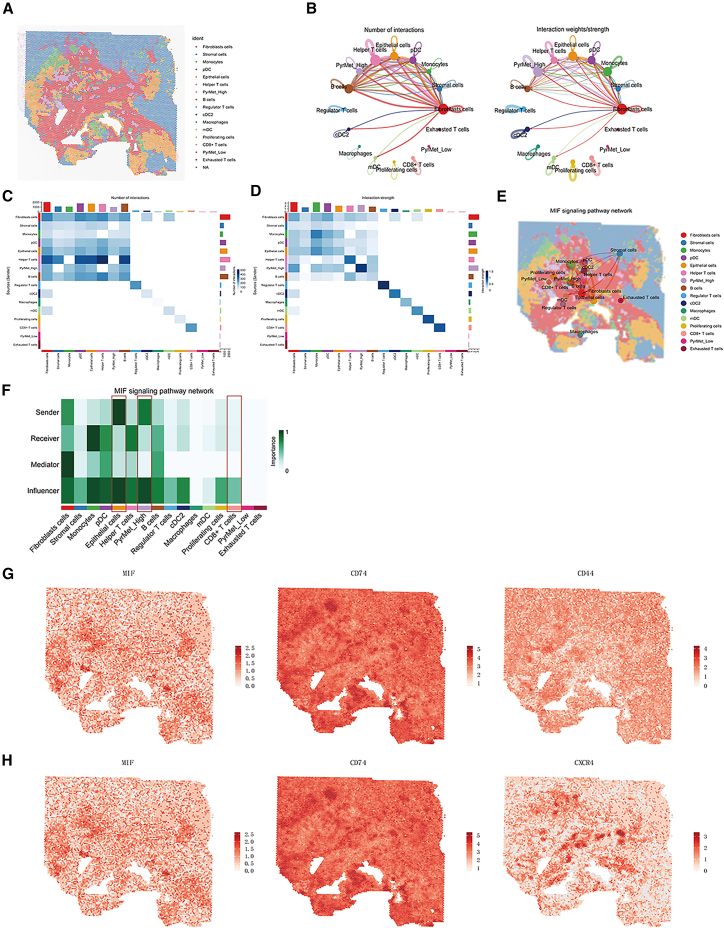
CellChat analysis of slice P6 (A) RCTD deconvolution visualization. (B) Number and strength of cell-cell communications. (C) Heatmap of communication numbers. (D) Heatmap of communication strength. (E) Visualization of MIF pathway interactions. (F) Heatmap of MIF pathway interactions. (G) Expression visualization of MIF-CD74-CD44 ligand-receptor pairs. (H) Expression visualization of MIF-CD74-CXCR4 ligand-receptor pairs.

**Figure 10:**
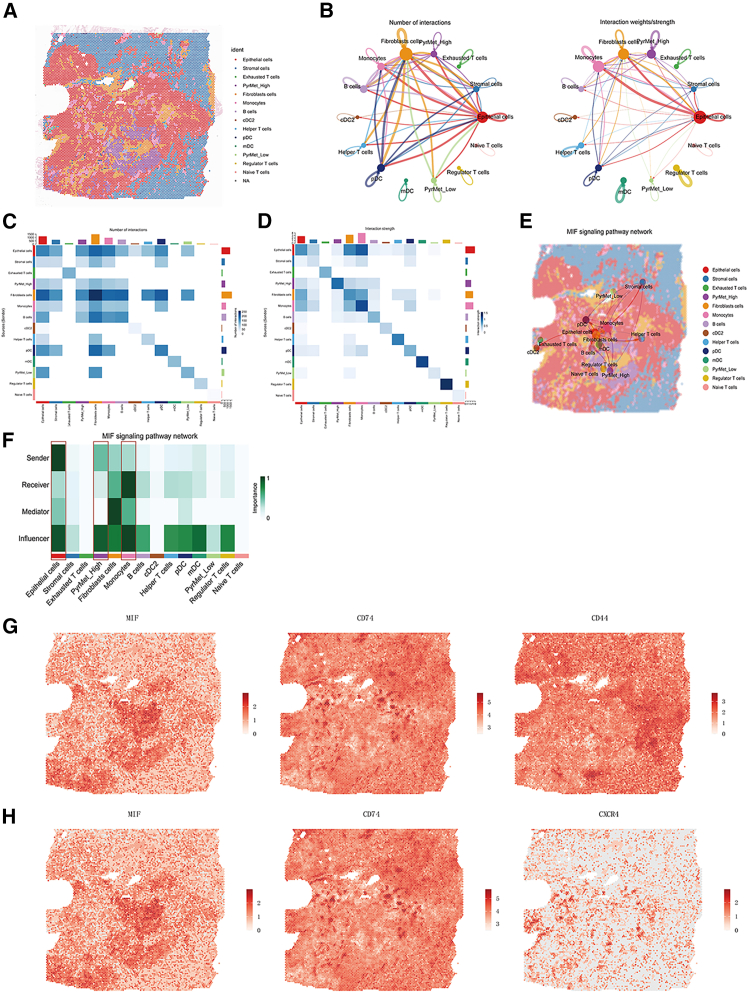
CellChat analysis of slice P4 (A) RCTD deconvolution visualization. (B) Number and strength of cell-cell communications. (C) Heatmap of communication numbers. (D) Heatmap of communication strength. (E) Visualization of MIF pathway interactions. (F) Heatmap of MIF pathway interactions. (G) Expression visualization of MIF-CD74-CD44 ligand-receptor pairs. (H) Expression visualization of MIF-CD74-CXCR4 ligand-receptor pairs.

Based on RCTD results of spatial transcriptomic (ST) slices, the major cell types identified included myeloid cells, epithelial cells, stromal cells, fibroblasts, PyrMet_Low, and PyrMet_High populations. Although the deconvolution yielded a relatively small number of PyrMet_Low cells inconsistent with the proportion in the single-cell matrix, this discrepancy may reflect biological differences, as the grouping was derived from scoring-based classification (Figures 9A and 10A).

In the two slices analyzed, intercellular signaling in P6 was predominantly driven by fibroblasts, whereas epithelial cells were the main signaling source in P4. Notably, high-pyrimidine metabolism tumor cells (PyrMet_High) exhibited significantly stronger communication intensity than low-pyrimidine metabolism tumor cells (PyrMet_Low) (Figures 9B–9D and 10B–10D).

Visualization of the MIF signaling pathway in ST slices revealed that MIF-mediated communication primarily occurred among stromal cells, fibroblasts, monocytes, and epithelial cells (Figures 9E, 9F, 10E, and 10F). Specifically, *MIF* was mainly expressed in high-pyrimidine metabolism tumor cells, while *CD74* was expressed in monocytes, *CD44* in fibroblasts, and *CXCR4* in monocytes/helper T cells (Figures 9G, 9H, 10G, and 10H).

### Metabolic pathway scoring and key pathway screening in LUAD patients

**Figure 11:**
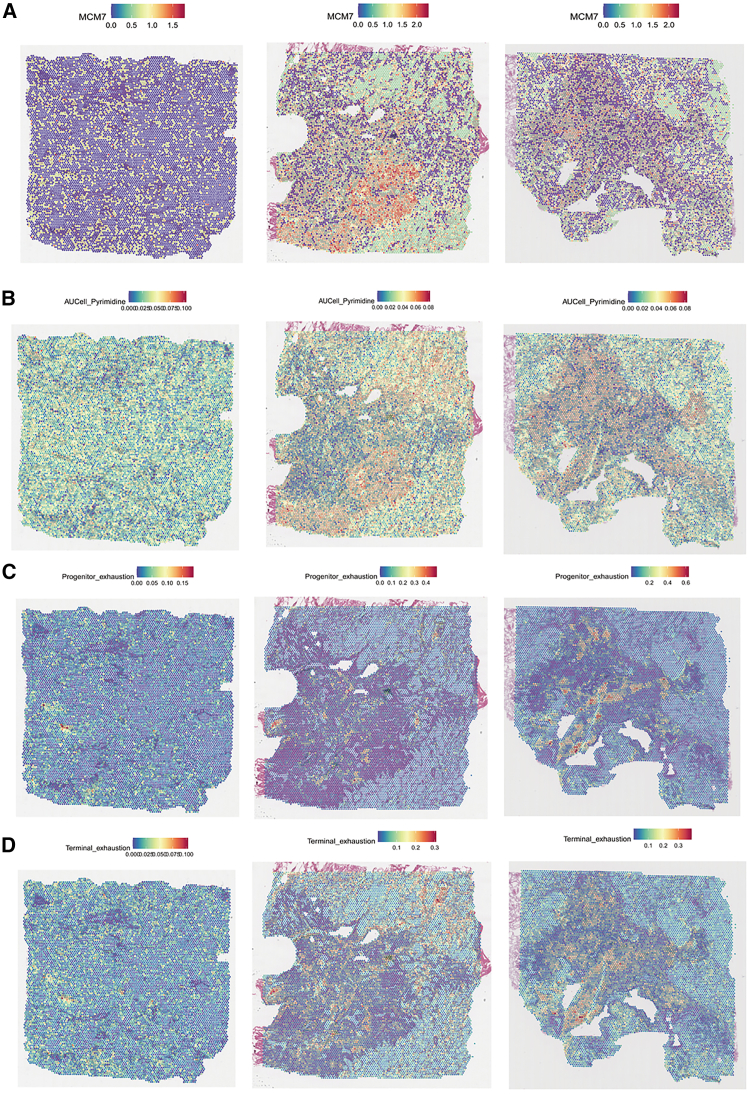
Spatial expression patterns and scoring in tissue sections (A) MCM7 expression pattern. (B) Pyrimidine metabolism scoring. (C) Initial T cell exhaustion scoring. (D) Terminal T cell exhaustion scoring.

Pyrimidine metabolism scores and T cell state scores were evaluated in one normal lung slice (P4_Nor) and two LUAD slices (P4 and P6). The results demonstrated spatial co-localization of *MCM7* expression with regions exhibiting high pyrimidine metabolism (Figures 11A and 11B). In contrast, the overlap between *MCM7* expression and regions with high T cell exhaustion scores was limited. Specifically, in slice P4, high pyrimidine metabolism regions showed no overlap with high T cell scoring areas, whereas in slice P6, partial overlap was observed between high pyrimidine metabolism regions and areas of terminal T cell exhaustion (Figures 11C and 11D).

### Single-cell analysis and pyrimidine metabolism scoring in LUAD patients

**Figure 12:**
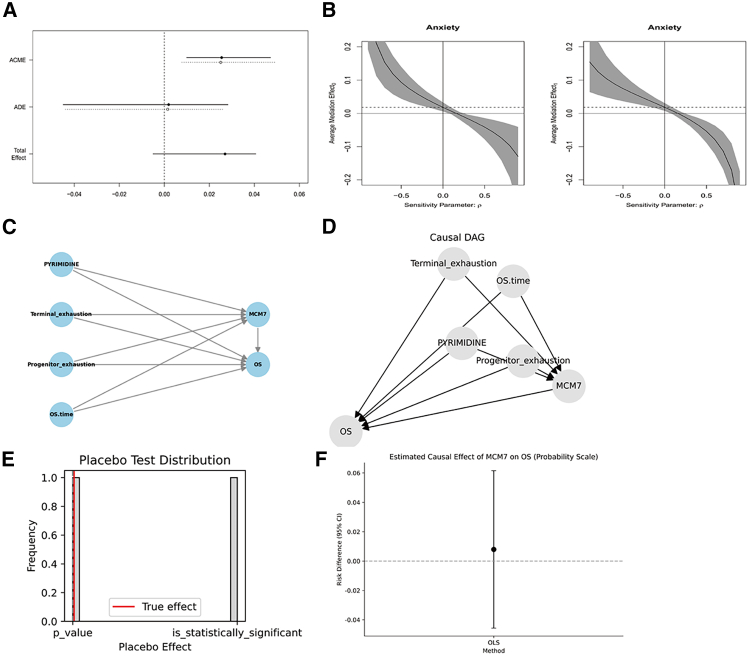
Mediation analysis and DoWhy causal analysis (A) Mediation model schematic. (B) RHO value curves. (C) Variable relationship diagram. (D) Causal directed acyclic graph (DAG). (E) Placebo analysis results. (F) Effect estimation plot.

Mediation analysis revealed that the average causal mediation effect (ACME) curve was located on the right side of the *y* axis, while the average direct effect (ADE) curve crossed the *y* axis, indicating that the independent variable *MCM7* had limited direct impact on OS outcomes, with the primary effect mediated through pyrimidine metabolism (Figure 12A). Larger absolute intercept values of the curves suggest higher model reliability (Figure 12B).

Using DoWhy causal analysis with *MCM7* expression as the exposure variable and OS as the outcome (Figures 12C and 12D), placebo substitution tests showed no substantial relationship between *MCM7* expression and OS outcomes (Figure 12E). However, the effect estimation plot suggested a potential, though statistically non-significant, association between *MCM7* expression levels and OS (Figure 12F).

### Silencing *MCM7* inhibits proliferation and migration of lung adenocarcinoma cells

**Figure 13:**
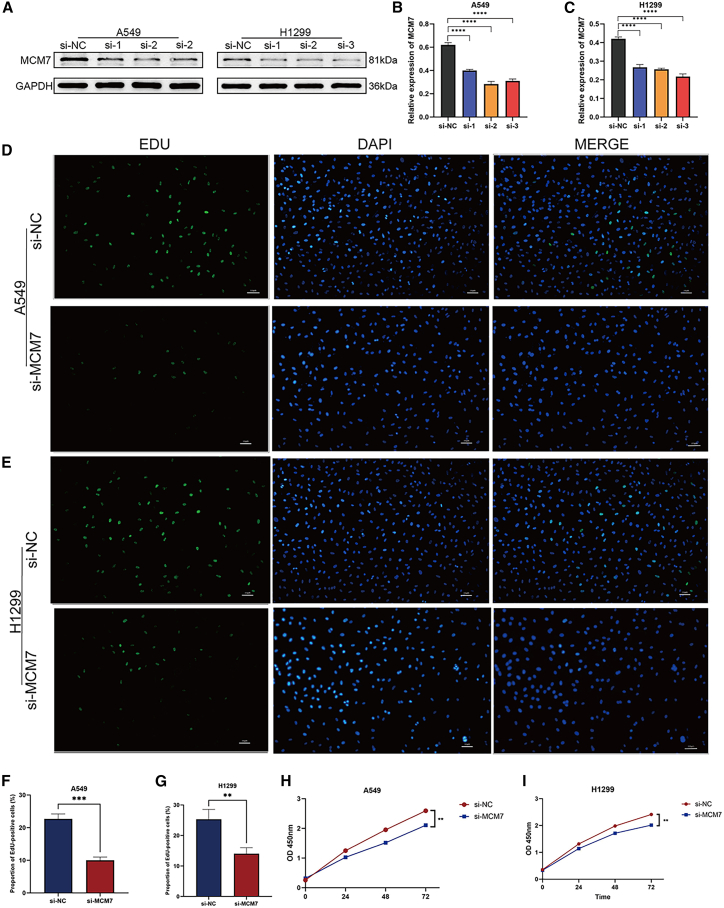
Knockdown of MCM7 inhibits the proliferation of lung adenocarcinoma cells (A) Western blot bands and (B and C) quantitative analysis showing the knockdown efficiency of MCM7 in A549 and H1299 cells after transfection with three specific MCM7 siRNAs (si-MCM7-1, -2, -3) or a negative control siRNA (si-NC). GAPDH was used as a loading control. (D–G) Representative images and quantitative analysis of EdU assays; proliferating cells show green fluorescence, and nuclei are counterstained with blue fluorescence. (H and I) Cell proliferation was detected by the CCK-8 assay. All data are from three independent biological replicate experiments and are presented as mean ± standard deviation. Scale bars, 100 μm. Statistical significance was determined by two-tailed Student’s *t* test. ∗*p* < 0.05, ∗∗*p* < 0.01, ∗∗∗*p* < 0.001, ∗∗∗∗*p* < 0.0001, ns indicates no significant difference.

**Figure 14:**
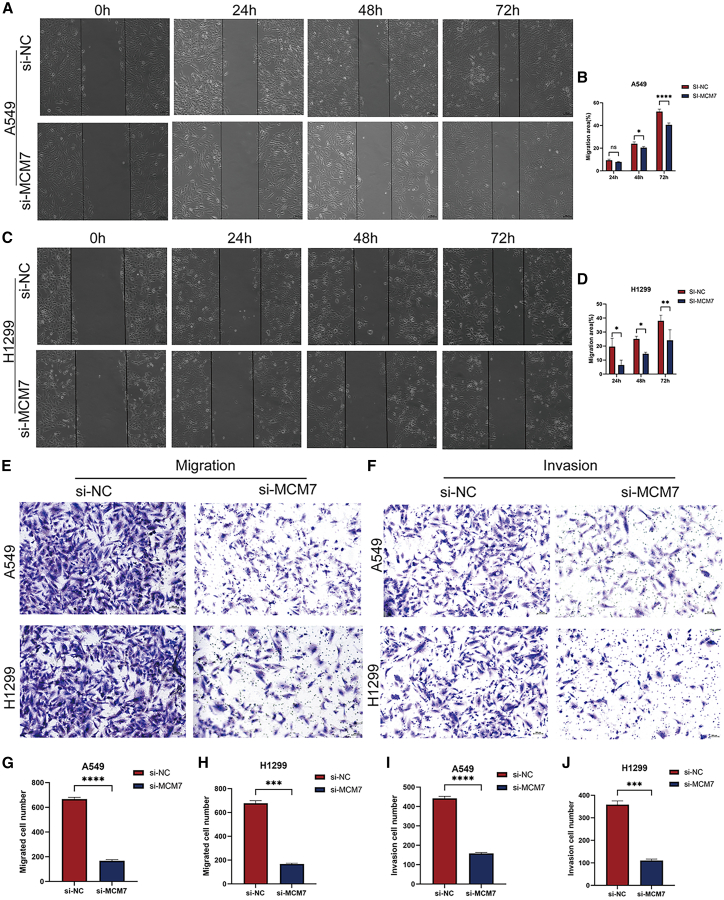
Knockdown of MCM7 inhibits the migration ability of lung adenocarcinoma cells (A–D) Representative images and quantitative analysis of the wound healing assay. (E–J) The effects of MCM7 deficiency on cell migration and invasion were investigated by Transwell assay and quantitative analysis. All data are from three independent biological replicate experiments and are presented as mean ± standard deviation. Scale bars, 100 μm. Statistical significance was determined by two-tailed Student’s *t* test. ∗*p* < 0.05, ∗∗*p* < 0.01, ∗∗∗*p* < 0.001, ∗∗∗∗*p* < 0.0001, ns indicates no significant difference.

To investigate the biological function of *MCM7* in lung adenocarcinoma, we transfected A549 and H1299 cells with si-*MCM7* to knock down its expression. Knockdown efficiency was verified by western blot analysis, which showed that all three si-*MCM7* sequences reduced MCM7 protein expression levels to varying degrees compared to the si-NC group (Figures 13A–13C). Among them, si-*MCM7*-2 demonstrated the most significant knockdown effect in A549 cells, while si-*MCM7*-3 showed the most pronounced effect in H1299 cells. Consequently, the most effective siRNA for each cell line was selected for all subsequent functional and mechanistic experiments. Results from 5-ethynyl-2′-deoxyuridine (EdU) and cell counting kit-8 (CCK-8) assays indicated that si-*MCM7* significantly inhibited cell proliferation viability compared to the si-NC group (Figures 13D–13I). Furthermore, wound healing and Transwell migration assays revealed that *MCM7* knockdown markedly attenuated cell migration capacity (Figures 14A–14J).

### *MCM7* specifically regulates key pyrimidine metabolic enzymes DHODH and UMPS and attenuates ERK signaling pathway phosphorylation

**Figure 15:**
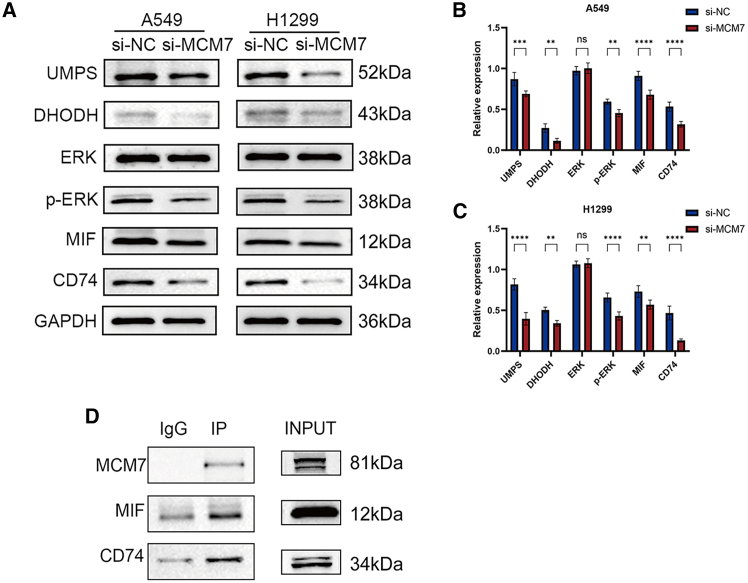
Downregulation of MCM7 affects key enzymes in the pyrimidine metabolism pathway, the ERK pathway, and the MIF signaling pathway (A–C) The effects of MCM7 deficiency on the expression of DHODH, UMPS, ERK, p-ERK, MIF, and CD74 in A549 and H1299 cells were analyzed and quantified by western blot. (D) Co-immunoprecipitation was performed in A549 and H1299 cells using an MCM7 antibody or control IgG, followed by western blot detection with MIF and CD74 antibodies. Input represents total cell lysate. GAPDH was used as a loading control. All data are from three independent biological replicate experiments and are presented as mean ± standard deviation. Statistical significance was determined by two-tailed Student’s *t* test. ∗*p* < 0.05, ∗∗*p* < 0.01, ∗∗∗*p* < 0.001, ∗∗∗∗*p* < 0.0001, ns indicates no significant difference.

Given that metabolic reprogramming is a hallmark of cancer, we further examined the effect of *MCM7* on the *de novo* pyrimidine synthesis pathway. Western blot analysis showed that knocking down *MCM7* reduced the protein levels of DHODH and UMPS. We subsequently explored potential downstream signaling pathways involved with *MCM7*. The results indicated that *MCM7* knockdown did not affect total ERK protein levels but significantly reduced the levels of its phosphorylated form, p-ERK (Figures 15A–15C).

### *MCM7* directly interacts with the MIF signaling pathway

To understand the potential mechanism by which *MCM7* participates in immunoregulation, we investigated its interaction with the MIF signaling pathway through co-immunoprecipitation. The results showed that the MCM7 antibody specifically co-precipitated both MIF and CD74 proteins, whereas this phenomenon was absent in the IgG control group (Figure 15D). This confirms the existence of a physical interaction between MCM7 and MIF and its receptor CD74 within the cells. Furthermore, knockdown of *MCM7* led to decreased protein expression levels of both MIF and CD74 (Figures 15A–15C).

## Discussion

Pyrimidine nucleotides serve as essential precursors for DNA and RNA synthesis, and their pathological upregulation is frequently observed in proliferating tumors.^27^ Consistent with previous reports, heightened pyrimidine metabolic flux accelerates tumor progression by enhancing cancer-cell proliferation and DNA-repair capacity.^28^^,^^29^ In this study, GSVA-based scoring revealed that LUAD patients with elevated pyrimidine-metabolism scores exhibited markedly worse prognosis, underscoring the central role of pyrimidine metabolism in cancer metabolic reprogramming. Independent LUAD cohort analyses have further corroborated that increased pyrimidine-metabolic signatures are associated with inferior clinical outcomes.^30^

By applying NMF clustering, we stratified LUAD into four molecular subtypes, among which cluster 4—defined by the highest pyrimidine-metabolism score—exhibited the most aggressive clinical course, thereby offering a metabolic dimension for LUAD taxonomy. Analogously, integrated multi-omics analyses of hepatocellular carcinoma have delineated two metabolic subtypes: a favorable-prognosis group driven by robust aerobic glycolysis, and an adverse-prognosis group hallmarked by hypoxia, lipid accumulation, hypermethylation of metabolic enzymes, and concomitant immune-pathway activation. These observations underscore the feasibility of exploiting metabolic heterogeneity as an independent prognostic framework.^31^ Importantly, the differentially expressed signature that defines cluster 4 constitutes a tractable target set for future therapeutic interventions.

We have further compared our approach with various prevalent prognostic model-building strategies (such as LASSO-Cox, random survival forest, and deep learning methods).^32^^,^^33^^,^^34^ In contrast to these methods, the StepCox[both] + RSF ensemble model we adopted maintains high predictive accuracy while simultaneously ensuring robustness in feature selection and model interpretability. This model demonstrated strong discriminative performance in both the training and test sets (AUC > 0.75). Moreover, the key genes it identified, such as *MCM7*, were subsequently functionally validated through single-cell and ST analyses.

In this study, T cell state scoring revealed a pronounced enrichment of exhausted T cell signatures in cluster 4. Consistent with our observations, afatinib has been reported to inhibit the rate-limiting enzyme CAD in *de novo* pyrimidine biosynthesis, thereby transiently limiting pyrimidine availability to CD8^+^ T cells and attenuating their proliferative capacity.^35^ Similarly, breast-cancer patients exhibiting elevated pyrimidine-metabolic scores display reduced intratumoral CD8^+^ T cell infiltration and unfavorable prognosis; concomitant upregulation of inhibitory receptors (PD-1, TIM-3, and LAG-3) further indicates functional T cell exhaustion.^36^ At single-cell resolution, we stratified tumors into PyrMetHigh and PyrMetLow subsets according to pyrimidine-metabolism scores. Notably, PyrMetHigh tumors displayed markedly augmented intercellular communication with CD8^+^ T cells, CD4^+^ T cells, and macrophages via MIF-dependent signaling, which correlated with a significant increase in exhaustion-associated markers. MIF, a pleiotropic cytokine central to immune and inflammatory responses, has been implicated in the pathogenesis of multiple malignancies, including breast, acute myeloid, colorectal, bladder, cervical, prostate, gastric, and lung cancers.^37^ Moreover, excessive MIF secretion by macrophages can precipitate life-threatening inflammatory cascades, and MIF itself serves as a cell-exhaustion marker for both CD8^+^ and CD4^+^ T cell compartments.^38^^,^^39^^,^^40^ Elevated dihydrofolate reductase (DHFR) activity drives intracellular accumulation of dihydrofolate, which directly engages the E3 ubiquitin ligase UBE4B and triggers JAK1 ubiquitination. This post-translational modification activates NF-κB signaling and accelerates epithelial-mesenchymal transition (EMT) in hepatocellular carcinoma.^41^ Concomitantly, MIF expands and activates myeloid-derived suppressor cells (MDSCs) and polarizes macrophages toward an M2-like phenotype, thereby establishing an immunosuppressive TME.^42^^,^^43^^,^^44^ The CD74-MIF axis is also implicated in the pathogenesis of multiple malignancies, including lung cancer,^45^ by driving tumor-associated macrophages (TAMs) toward an immunosuppressive state and inducing secretion of IL-10 and ARG1, which directly inhibit CD8^+^ T cell activity and enforce exhaustion.^46^ Moreover, neuroblastoma-secreted MIF induces IFN-γ-mediated apoptosis of activated T cells, facilitating immune evasion.^43^ Conversely, genetic ablation of MIF and its homolog D-DT impairs glucose uptake, diminishes pentose phosphate pathway flux, reduces NADPH-dependent glutathione regeneration, and elevates intracellular hydrogen peroxide.^47^^,^^48^ Nevertheless, the mechanistic interplay between pyrimidine metabolism and TME remodeling remains incompletely understood.

In our integrative analysis of 33 prognostic marker genes, *MCM7* was identified as a network hub gene whose overexpression correlated positively with CD8^+^ T cell infiltration and *MIF* signaling, thereby delineating a MCM7-MIF-CD8^+^ T cell regulatory axis in LUAD. High *MCM7* expression was independently associated with shortened OS and exhibited coordinated upregulation with the *MIF* pathway at single-cell resolution. *MCM7*, an essential component of the DNA replication pre-initiation complex, orchestrates cell-cycle progression and DNA synthesis.^49^ Aberrant *MCM7* expression drives tumorigenesis and progression across multiple malignancies, and its pharmacologic inhibition disrupts DNA replication to suppress lung cancer growth.^50^^,^^51^ In Saccharomyces cerevisiae, *MCM7* down-regulation represses genes involved in *de novo* pyrimidine biosynthesis, underscoring its role in nucleotide homeostasis.^52^ In lung squamous cell carcinoma, *MCM7* expression levels correlate with infiltrating B cells, T cells, TAMs, M1 macrophages, T follicular helper cells, and Th17 cells.^53^

As a core component of the MCM2-7 complex, *MCM7* plays a canonical role in the initiation of DNA replication.^54^ Research has found that in bladder cancer stem-like cells, *MCM7* expression is not downregulated with cell cycle slowing but instead maintains cell stemness by enhancing autophagic flux.^55^ Studies in hepatocellular carcinoma have also shown that *MCM7* promotes tumor proliferation and progression by regulating the MAPK-cyclin D1 signaling pathway.^56^ These findings collectively suggest that *MCM7* may act as a central node integrating multiple processes such as replication stress response, metabolic reprogramming, and immune regulation. While maintaining replication integrity, *MCM7* may influence the metabolic adaptability of tumor cells by regulating the expression of key pyrimidine metabolism enzymes (such as DHODH); furthermore, its direct interaction with the identified MIF-CD74 complex further implies a non-canonical function for *MCM7* in regulating immune evasion within the TME. Additionally, the attenuation of ERK signaling upon *MCM7* knockdown may serve as a convergence point, coordinating its dual regulation of metabolic pathways and immune signaling.

Our study, integrating ST with causal inference frameworks, reveals that regions of high *MCM7* expression exhibit significant spatial co-localization with elevated pyrimidine metabolism activity. However, *MCM7* expression demonstrates no substantial association with T cell functional states. Mediation analysis identifies pyrimidine metabolism as the principal driver of OS outcomes, with *MCM7* exerting only minimal direct effects. Consistent with this, DoWhy-based causal inference analysis shows that the ordinary least squares (OLS) estimate centers above the *x* axis. Given the binary nature of the OS endpoint, these results indicate that *MCM7* expression exerts a modest but positive effect on OS, wherein elevated *MCM7* levels promote adverse clinical outcomes primarily through pyrimidine metabolic reprogramming rather than through direct mechanisms.

### Conclusions

We integrated bulk and single-cell transcriptomic data with machine-learning algorithms to comprehensively dissect the interplay between pyrimidine metabolism and the immune microenvironment in LUAD. Our findings reveal that elevated pyrimidine anabolism is associated with T cell exhaustion and altered intercellular communication, leading to adverse clinical outcomes. We provide the first evidence that *MCM7*-mediated pyrimidine metabolic reprogramming enhances crosstalk among malignant cells, CD8^+^ and CD4^+^ T cells, and macrophages while simultaneously amplifying exhaustion signatures in both T cell subsets, thereby accelerating LUAD progression. These results delineate a *MCM7*-pyrimidine immunity axis and identify combinatorial metabolic-immune targets for future therapeutic strategies.

### Limitations of the study

While our integrated multi-omics analyses and subsequent experimental validation have established *MCM7* as a critical regulator linking pyrimidine metabolism to *MIF*-mediated immunosuppression, several aspects require further investigation. First, the clinical applicability of our constructed 9-gene prognostic signature remains limited by its complexity, necessitating future refinement into a more clinically tractable core gene set. Additionally, further validation is needed to directly demonstrate the functional impact of *MIF* signaling on CD8^+^ T cell exhaustion through co-culture systems and to evaluate how *MCM7* expression regulates tumor growth and immune microenvironment dynamics using immunocompetent mouse models. These investigations will provide crucial evidence for the biological significance of this pathway and lay the groundwork for developing combination therapeutic strategies targeting the high-pyrimidine metabolism subtype.

## Resource availability

### Lead contact

Further information and requests for resources and reagents should be directed to and will be fulfilled by the lead contact, Dr. Lou Zhong (tdfyzl@ntu.edu.cn).

### Materials availability

This study did not generate new unique reagents.

### Data and code availability

• All data used in this study were obtained from publicly available databases. The TCGA-LUAD dataset was sourced from the GDC portal (https://portal.gdc.cancer.gov), while the validation cohort GSE68465, ST database (GSE307534), and the single-cell datasets (GSE148071 and GSE189357) were obtained from the GEO database (https://www.ncbi.nlm.nih.gov/geo). Clinical and survival data were accessed via the UCSC Xena platform (https://xena.ucsc.edu). All processed data are publicly accessible through the above-mentioned accession numbers from the original databases.
• This study did not generate any new custom code.
• Any additional information required to reanalyze the data reported in this paper is available from the lead contact upon request.

## Acknowledgments

This work was supported by the Natural Science Research Project of Nantong Science and Technology Bureau (no. MS12020029).

## Author contributions

M.Y.L., writing – original draft, writing – review and editing, and validation; H.Q.L., investigation and writing – review and editing; S.H.X., visualization and writing – review and editing; Y.K.Z., investigation and writing – review and editing; M.Y., software and writing – review and editing; J.H.S., software and writing – review and editing; L.Z., supervision and writing – review and editing.

## Declaration of interests

The authors declare no competing interests.

## STAR★Methods

### Key resources table

**Table:** 

REAGENT or RESOURCE	SOURCE	IDENTIFIER
**Antibodies**		

Anti-MCM7 antibody	Proteintech	Cat#11225-1-AP
Anti-CD74 antibody	Proteintech	Cat#66390-1-Ig
Anti-MIF antibody	Proteintech	Cat#20415-1-AP
Anti-UMPS antibody	Proteintech	Cat#14830-1-AP
Anti-DHODH antibody	Proteintech	Cat#14877-1-AP
Anti-β-actin antibody	Proteintech	Cat No. 60009-1-Ig
Anti-GAPDH antibody	Proteintech	Cat No. 60004-1-Ig
Normal rabbit IgG	Proteintech	Used as negative control (Co-IP)
HRP-conjugated secondary antibody	Proteintech	Cat No. SA00001-11

**Biological samples**		

Human LUAD tumor tissue	Affiliated Hospital of Nantong University	N/A
Paired adjacent normal lung tissue	Affiliated Hospital of Nantong University	N/A

**Chemicals, peptides, and recombinant proteins**		

RPMI-1640 medium	Gibco	A4192301
Dulbecco’s Modified Eagle Medium (DMEM)	Gibco	A4192101
Fetal Bovine Serum (FBS)	Cytiva	SH30406.06
Lipofectamine™ 3000 transfection reagent	Thermo Fisher Scientific	L3000015
CCK-8 solution	Dojindo Laboratories	CK04-500T
EdU assay kit	Beyotime Biotechnology	C0071S
4% paraformaldehyde	Solarbio	P1110
Triton X-100	Sigma-Aldrich	X100-100 ML
Matrigel	BD Biosciences	356234
Transwell chambers (8 μm pore size)	Corning	3428
0.1% crystal violet solution	Servicebio	G1014-50 ML
Nondenaturing lysis buffer	Cell Signaling Technology	9803S
RIPA buffer	Servicebio	G2002-100 ML
Protease inhibitor cocktail	Roche	11697498001
BCA protein assay kit	Thermo Fisher Scientific	A55861
10% SDS-PAGE gels	Epizyme	PG212
PVDF membranes	Beyotime Biotechnology	FFP22
ECL substrate	Bio-Rad	Bio-Rad

**Critical commercial assays**		

TRIzol reagent	Invitrogen	Cat#15596018
HiScript II RT SuperMix	Vazyme	R223-01
SYBR Green Master Mix	Thermo Fisher	A46109
Protein A/G agarose beads	Cell Signaling Technology	37478P

**Deposited data**		

TCGA-LUAD bulk RNA-Seq profiles	Genomic Data Commons (GDC)	https://portal.gdc.cancer.gov
GEO expression profiles (GSE68465, GSE148071, GSE189357)	Gene Expression Omnibus (GEO)	https://www.ncbi.nlm.nih.gov/geo

**Experimental models: Cell lines**		

H1299	American Type Culture Collection (ATCC)	ATCC CRL-5803
A549	American Type Culture Collection (ATCC)	ATCC CCL-185
BEAS-2B	American Type Culture Collection (ATCC)	ATCC CRL-9609

**Oligonucleotides**		

si-*MCM7*-1 (sense):5′-GCUCCAGAUUCAUCAAAUUTT-3′	This paper	N/A
si-*MCM7*-1 (antisense):5′-AAUUUGAUGAAUCUGGAGCTT-3′	This paper	N/A
si-*MCM7*-2 (sense):5′-GGGUGUGUGCUGCAUUGAUTT-3′	This paper	N/A
si-*MCM7*-2 (antisense):5′-AUCAAUGCAGCACACACCCTT-3′	This paper	N/A
si-*MCM7*-3 (sense):5′-GGAUGUGGUGGAGAAAGAATT-3′	This paper	N/A
si-*MCM7*-3 (antisense):5′-UUCUUUCUCCACCACAUCCTT-3′	This paper	N/A
*MCM7* qRT-PCR primer (F):5′-GGGAGGAGTGAGGCAAGTT-3′	This paper	N/A
*MCM7* qRT-PCR primer (R):5′-ATGTTGATGTGCCCCGGAT-3′	This paper	N/A
β-actin qRT-PCR primer (F):5′-CATGTACGTTGCTATCCAGGC-3′	This paper	N/A
β-actin qRT-PCR primer (R):5′-CTCCTTAATGTCACGCACGAT-3′	This paper	N/A

**Software and algorithms**		

GraphPad Prism	GraphPad Software	https://www.graphpad.com/scientific-software/prism/
R software	R Foundation for Statistical Computing	https://www.r-project.org/
Python	Python Software Foundation	https://www.python.org/
ImageJ	National Institutes of Health (NIH)	https://imagej.nih.gov/ij/
QuantStudio 6 instrument software	Thermo Fisher Scientific	https://www.thermofisher.cn/cn/zh/home/technical-resources/software-downloads/quantstudio-6-7-pro-real-time-pcr-system.html

### Experimental model and study participant details

#### Human tissue samples

This study enrolled 9 patients with lung adenocarcinoma (LUAD) who underwent surgical treatment at the Affiliated Hospital of Nantong University between October and November 2022. All cases were confirmed by pathological examination. During surgery, tumor tissue and its paired adjacent normal tissue (taken from a location more than 5 cm from the tumor margin) were collected from each patient. Inclusion criteria required that patients had not received any systemic anticancer therapy (such as chemotherapy, radiotherapy, or targeted therapy) prior to surgery. The collected tissue samples were immediately frozen in liquid nitrogen and then transferred to a -80°C ultra-low temperature freezer for storage and subsequent analysis. This study strictly adhered to ethical guidelines and was approved by the Ethics Committee of Nantong University Affiliated Hospital (Approval Number: 2021-L142). And all participating patients signed written informed consent forms.

#### Cells

The LUAD cell lines H1299 and A549, along with the normal bronchial epithelial line BEAS-2B, were obtained from the American Type Culture Collection (ATCC). H1299 cells were maintained in RPMI-1640 (Gibco) supplemented with 10% fetal bovine serum (FBS; Cytiva). A549 and BEAS-2B cells were cultured in Dulbecco’s Modified Eagle Medium (DMEM, Gibco) containing 10% FBS. All cultures were incubated at 37°C with 5% CO_2_ in a humidified atmosphere (Thermo Scientific).

### Method details

#### Data acquisition and processing

Bulk RNA-Seq profiles of 576 LUAD tumors (TCGA-LUAD) were retrieved from the Genomic Data Commons (https://portal.gdc.cancer.gov); the GSE68465 series (n = 442) served as an external validation cohort from the Gene Expression Omnibus (https://www.ncbi.nlm.nih.gov/geo). For single-cell analyses, we integrated two public scRNA-seq datasets (GSE148071, 42 patients; GSE189357, 9 patients). Corresponding clinical and survival data were obtained via UCSC Xena (https://xena.ucsc.edu) and filtered to retain samples with complete follow-up for downstream analyses. For spatial transcriptomics (ST) analysis, 7 ST slices from LUAD patients were obtained from the GEO dataset GSE307534. Bulk RNA-seq data from the UCSC Xena preprocessed dataset served as the training set. Batch effects were not considered for the independent validation analysis.The batch effects in the transcriptome data were removed using the removeBatchEffect function from the limma package.

#### GSVA scoring of KEGG pathways

The R package msigdbr (Version 7.5.1) was used to obtain KEGG pathway information(Species: Homo sapiens), and the R package GSVA (Version 2.0.5) was used for GSVA scoring.

#### Survival analysis

The R package survival (Version 3.8-3) was used to perform survival analysis on the TCGA matrix. Univariate COX regression was conducted for 33 marker genes based on survival time, and the matrix was divided into high- and low-risk groups according to the median risk. The R package survminer (Version 0.5.0) was employed to plot the K-M curves. A multivariate Cox regression model was constructed using the survival package in R, with the model formula as follows: Surv(OS.time, OS) ∼ Risk Score + Age + Gender + Stage. Here, OS.time represents overall survival time, and OS represents survival status (0 for alive, 1 for deceased).

#### NMF (non-negative matrix factorization) analysis

The R package NMF (Version 0.28) was used for non-negative matrix factorization analysis. The Rank value selection was confirmed using the following parameters: Residual Sum of Squares (RSS), Cophenetic Correlation Coefficient, Silhouette Score, Dispersion, and Sparseness.

#### Differential expression analysis and enrichment analysis

The R package Limma (Version 3.26.1) was used for differential analysis, with parameters set to |Log2 Fold Change| > 0.5 and FDR < 0.05. The R package “ggplot2 (Version 3.5.1)” was used to visualize the differential expression results, with the top 10 genes in |Log2 FC| among the up- and down-regulated genes labeled separately. The R package BioEnricher (Version 0.1.0) was used for GO enrichment analysis, KEGG enrichment analysis, and GSEA enrichment analysis and visualization.

#### Single-cell analysis

The Seurat (version 5.2.1) software package was used for preliminary analysis of single-cell data. Only genes expressed in at least 0.1% of cells were included in the analysis. Then, the expression counts for each cell were log-normalized and scaled (by a factor of 10,000). Variable genes were detected using the VST method, and the top 2,000 genes were selected for cluster analysis. The R package harmony (Version 1.2.3) was used for batch normalization. Principal component analysis (PCA) was then performed on the variable genes, and the top 30 principal components were selected for downstream analysis. Cells were clustered using the FindNeighbors and FindClusters functions in Seurat, with dimensions set to 1:30 and resolution set to 0.3. Cell annotation was performed manually, with cell marker gene sources.^1^^,^^2^ Visualization analysis was performed using the R packages “SCP (version: 0.5.6)” and “Nebulosa (version: 1.16.0)” to plot single-cell UMAP clustering diagrams and density plots.

#### Copy number variation analysis

Copy number variation analysis was performed using infercnvpy (Version 0.4.5). All epithelial cells were retained, and 2,000 cells were randomly selected from each type of normal cell as normal cell references. Based on the CNV inference results and clustering results, epithelial cells were identified as normal epithelial cells and malignant epithelial cells.

#### Single-cell metabolic pathway scoring and differential expression analysis

The R package scMetabolism (Version 0.2.1) was used for single-cell metabolic pathway scoring. Based on the scoring results, malignant epithelial cells were divided into two groups: pyrimidine metabolism high (PyrMet_High) and low (PyrMet_Low). Differentially expressed genes were calculated using the FindAllMarkers function with min.pct = 0.25 and logfc.threshold = 0.25.

#### Machine learning for constructing a risk scoring model

The R package Mime1 (Version 0.0.0.9000) was used to construct a risk scoring model using machine learning methods, the package incorporates ten machine learning algorithms for constructing prognostic models, including LASSO, Ridge, Elastic Net, Random Survival Forest, CoxBoost, Stepwise Cox (StepCox[forward]/StepCox[backward]), plsRcox, SuperPC, RSF + LASSO, and Enet + CoxBoost. Each algorithm undergoes internal 5-fold cross-validation, with external performance evaluated through 100 bootstrap resamplings to estimate the C-index. Based on the model, genes common to both the training set and the test set were selected as feature genes, and unicox.filter.for.candi = T, mode = ALL, to further screen genes for training. We then used 10 machine learning algorithms and their combinations, totaling 101 methods, to construct the risk scoring model, while calculating the C-index and predictive AUC values for each model. The best model was selected for subsequent analysis.

#### T-cell state scoring

The R package TCellSI was used to assess the T-cell state of LUAD patients and compare the differences in T-cell state between Cluster 4 patients and other patients. Likewise, the R package TCellSI was employed to evaluate the T-cell state in LUAD patients, specifically comparing T-cell exhaustion levels between LUAD tissue sections and normal tissue sections.

#### Immune infiltration analysis

The R package CIBERSORT (version 0.1.0) was used for immune infiltration analysis of LUAD patients, with 1,000 permutations.

#### Cell communication analysis

The R package CellChat (version 1.6.1) was utilized for cell-cell communication analysis of single-cell data. The matrix was split into PyrMet_High and PyrMet_Low matrices, from which CellChat objects were generated separately. Using the built-in “SecretedSignaling” ligand-receptor database, communication probabilities were computed via computeCommunProb, followed by calculation of signaling flow centrality for each cell population through netAnalysis_computeCentrality. Visualization was performed using functions such as netAnalysis_signalingRole_heatmap. We further compared differences in key signaling pathways between the PyrMet_High and PyrMet_Low groups and visualized interaction patterns of ligand-receptor pairs.

For spatial transcriptomics (ST) analysis, three methods—SpaGene, CellChat, and Commot—were employed to conduct cell-cell communication analysis on HCC tissue sections. SpaGene identifies spatially variable genes and co-localized gene pairs from spatial omics data, enabling analysis of ligand-receptor (LR) interactions in tissue sections through LR databases. Commot constructs cell-cell communication (CCC) networks for ligand-receptor pairs within a spatial distance constraint of 500 using collective optimal transport and analyzes information flow directions of specified LR pairs.

#### Correlation analysis

Pearson correlation analysis and visualization were performed using the R package corrplot (Version 0.95).

#### scTenifoldKnk

PyrMet_High cells were selected to extract the expression matrix. The R package scTenifoldKnk was used to simulate the knockout of the MCM7 gene. scTenifoldKnk compares the knocked-down scGRN with the wild-type (WT) scGRN to identify differentially regulated genes, referred to as virtual knockout perturbation genes, for assessing the impact of gene knockout and revealing gene function in the analyzed cells. Parameters were set as follows: nc_nNet = 10, nc_nCells = 500, nc_nComp = 30.

#### Spatial transcriptomics

The dataset GSE307534 was downloaded from the GEO database. The first six LUAD slices (P1–P6) and one adjacent normal tissue slice from patient P4 (P4_Nor), totaling 80,000 spots, were selected. After sequential reading, data were normalized using SCTransform, integrated, and batch-corrected with RunHarmony. The single-cell matrix mentioned above served as the deconvolution reference. The RCTD algorithm was applied to deconvolute spots, determining the cell type of each spot based on the highest proportional cell type.

#### AUcell

Pyrimidine metabolism-related gene sets were downloaded from the MSIGDB website. AUcell was used to score spots in the tissue sections, and results were visualized with the SpatialFeaturePlot function.

#### Mediation analysis

A model was constructed with *MCM7* expression as the independent variable, patient survival status as the outcome, and pyrimidine metabolism score as the mediator. T-cell state scores and survival time were included as confounding variables. The analysis was performed with 1,000 iterations.

#### DoWhy causal analysis

Causal inference was conducted using Python 3.10 and the DoWhy 0.14 framework. A causal directed acyclic graph (DAG) was constructed based on domain knowledge, assuming all covariates as confounders. The average treatment effect (ATE) was identified via the backdoor criterion. Ordinary least squares (OLS) regression was used to estimate the causal effect of the continuous exposure variable “*MCM7*” on the binary outcome “OS,” with all covariates included to control for confounding. To assess robustness, three native refutation tests were implemented: data subset (80%), placebo treatment, and random common cause. Bootstrap resampling (500 iterations) was used to derive 95% confidence intervals.

#### CWGCNA

Four outcomes were selected: disease occurrence, gender, survival status, and survival time. Using topvaricancetype = “sd”, the top 10,000 genes with the highest standard deviation were retained for subsequent network construction. Bootstrap resampling was performed 1,000 times to calculate mediation effect consistency. For modules identified as “causally driven,” module eigengenes were further extracted for functional enrichment analysis.

#### siRNA transfection

Three specific small interfering RNAs (si-*MCM7*) targeting the human *MCM7* gene and one negative control siRNA (si-NC) were designed and synthesized by Gema Pharmaceutical Technology Co., Ltd. (Shanghai, China). The sequence information is as follows: si-*MCM7*-1 sense, 5’-GCUCCAGAUUCAUCAAAUUTT-3’ and antisense, 5’-AAUUUGAUGAAUCUGGAGCTT-3’; si-*MCM7*-2 sense, 5’-GGGUGUGUGCUGCAUUGAUTT-3’ and antisense, 5’-AUCAAUGCAGCACACACCCTT-3’; si-*MCM7*-3 sense, 5’-GGAUGUGGUGGAGAAAGAATT-3’ and antisense, 5’-UUCUUUCUCCACCACAUCCTT-3’. When cells reached 60-70% confluence in 6-well plates, transfection was performed using Lipofectamine™ 3000 transfection reagent according to the manufacturer's instructions. Each well received 125 pmol siRNA and 5 μL Lipofectamine™ 3000 reagent. Cells were harvested 48 hours post-transfection.

#### CCK-8 assay

Transfected cells were seeded into 96-well plates at a density of 1×10^3^ cells per well, with 6 replicate wells per group. At 0, 24, 48, and 72 hours after seeding, 10 μL of CCK-8 (Dojindo Laboratories) solution was added to each well. The plate was returned to the incubator for an additional 2 hours. Subsequently, the absorbance at 450 nm wavelength was measured using a microplate reader.

#### EdU assay

Cells transfected for 48 hours were seeded into 96-well plates. After 24 hours of culture, 50 μM EdU working solution was added, followed by incubation for another 2 hours. The medium was discarded, and cells were fixed with 4% paraformaldehyde for 15 minutes, followed by permeabilization with 0.5% Triton X-100 for 20 minutes. Apollo staining reaction solution was added and incubated for 30 minutes in the dark. Finally, cell nuclei were counterstained with Hoechst 33342. Cells were observed under a fluorescence microscope, and five random fields were captured. The percentage of EdU-positive cells (green fluorescence) relative to the total number of cells (blue fluorescence) was calculated. (Beyotime Biotechnology).

#### Wound healing assay

Transfected cells were seeded into 6-well plates at a density of 5×10^5^ cells per well and cultured in a 37°C, 5% CO_2_ incubator until confluence exceeded 90%. A uniform scratch/wound was created in the cell monolayer using a sterile 200 μL pipette tip, perpendicular to pre-marked lines on the bottom of the plate. Cells were gently washed twice with pre-warmed PBS to remove detached cell debris, and then the medium was replaced with serum-free medium. Images of the wound area were taken at the same pre-marked locations under an inverted microscope at 0, 24, 48, and 72 hours post-scratching. The wound area was measured using ImageJ software, and the wound healing rate was calculated using the following formula: Wound Healing Rate (%) = [1 - (Wound area at indicated time point / Wound area at 0 hour)] × 100%.

#### Cell invasion and migration assay

Cell invasion and migration assays were performed using Transwell chambers (polycarbonate membrane, 8 μm pore size, Corning). For the cell invasion assay, the upper chambers were first coated with 50 μL of Matrigel (BD Biosciences) and incubated at 37°C for 1 h to solidify. For the cell migration assay, no Matrigel was added. Cells transfected for 48 hours were resuspended in serum-free medium, counted, and approximately 5×10^4^ cells in 100 μL were seeded into the upper chamber. The lower chamber was filled with 600 μL of complete medium containing 20% FBS as a chemoattractant. The chambers were placed in a 37°C, 5% CO_2_ incubator for 24 hours. Subsequently, the non-migrated/non-invaded cells on the upper surface of the membrane were carefully removed with a cotton swab. Cells that had migrated/invaded to the lower surface of the membrane were fixed with 4% paraformaldehyde for 15 minutes and stained with 0.1% crystal violet solution for 20 minutes. Five random fields per membrane were imaged and counted under an optical microscope (Nikon Eclipse E100). The average number of cells from the five fields was used for statistical analysis.

#### Co-immunoprecipitation (Co-IP)

To detect endogenous protein interactions, cells were cultured in 10-cm dishes until they reached 80-90% confluence. Subsequently, the cells were lysed on ice using a nondenaturing lysis buffer. The cell lysates were incubated with 3 μg of the corresponding antibody (anti-MCM7 antibody or normal rabbit IgG) at room temperature for 2 hours. Following this, 40 μL of thoroughly resuspended Protein A/G agarose beads were added, and the incubation was continued at 4°C for 4 hours. The agarose beads were pelleted by brief centrifugation and washed five times with ice-cold IP lysis buffer. Finally, 1× SDS loading buffer was added to the pellets and boiled for 10 minutes to dissociate the immunoprecipitated complexes from the beads. The samples were then analyzed by Western Blot to detect MIF and CD74 proteins.

#### Quantitative real-time PCR (qRT-PCR)

Total RNA was extracted using TRIzol (Invitrogen) and reverse-transcribed with HiScript II RT SuperMix (Vazyme). qRT-PCR was performed in triplicate with SYBR Green Master Mix (Thermo Fisher) on a QuantStudio 6 instrument. Primers: *MCM7* (F: 5′-GGGAGGAGTGAGGCAAGTT-3′, R: 5′-ATGTTGATGTGCCCCGGAT-3′) and β-actin (F: 5′-CATGTACGTTGCTATCCAGGC-3′, R: 5′-CTCCTTAATGTCACGCACGAT-3′). Relative expression was calculated by the 2ˆ(–ΔΔCt) method with β-actin as reference.

#### Western blotting

Cells or tissues were lysed in RIPA buffer containing 1× protease inhibitor cocktail. After 30 min on ice, lysates were centrifuged (12 000 × g, 15 min, 4°C) and protein concentrations determined by BCA assay (Thermo). Equal amounts (30 μg) were separated on 10% SDS-PAGE gels, transferred to PVDF membranes, and blocked with 5% non-fat milk (1 h, RT). Membranes were incubated overnight at 4°C with anti-MCM7 (1:5 000; 11225-1-AP, Proteintech), anti-CD74, anti-MIF (1:1,000; 20415-1-AP, Proteintech), anti-UMPS (1:1,000; 14830-1-AP, Proteintech), anti-DHODH (1:2,000; 14877-1-AP, Proteintech), anti-GAPDH (1:1,0000; 60004-1-Ig, Proteintech)and anti-β-actin (1:20 000; 66009-1-Ig, Proteintech), followed by HRP-conjugated secondary antibodies (1:5 000, 2 h, RT). Bands were visualized with ECL substrate (Bio-Rad) and quantified using ImageLab, normalized to β-actin.

### Quantification and statistical analysis

All data were processed, analyzed, and plotted using GraphPad Prism (v10), R (v4.4.2), and Python (v3.9.21) software. Comparisons between groups were performed using two-tailed Student’s t-test or two-way analysis of variance (ANOVA), as appropriate based on data characteristics. Survival analysis was conducted using the Kaplan-Meier method and Log-rank test. Correlation analysis employed the Pearson correlation coefficient. The performance of machine learning models was evaluated using the concordance index (C-index) and the area under the receiver operating characteristic curve (AUC). All causal inference and mediation analyses underwent corresponding robustness tests (e.g., bootstrapping, placebo tests). Continuous variable data are presented as mean ± standard deviation (mean ± SD). All *in vitro* experiments were performed with at least three independent biological replicates. Statistical significance was defined as p < 0.05. In the figures, significance is denoted by the following symbols: ns (p > 0.05), ∗ (p < 0.05), ∗∗ (p < 0.01), ∗∗∗ (p < 0.001), ∗∗∗∗ (p < 0.0001).

#### Ethical approval

This study strictly adhered to ethical guidelines and was approved by the Ethics Committee of Nantong University Affiliated Hospital (Approval Number: 2021-L142). And all participating patients signed written informed consent forms.

### Additional resources

This work is not part of/involves a clinical trial.
