# Supplementary material for: Multi-omics integration reveals that pyrimidine metabolism in lung adenocarcinoma drives an immunosuppressive microenvironment
Source: iScience. 2026 Mar 18;29(4):115326. doi: 10.1016/j.isci.2026.115326 (PMC13084340; doi:10.1016/j.isci.2026.115326)
Supplement: Document S1. Figures S1–S5 [file mmc1.pdf]

iScience, Volume *29*

Supplemental information

Multi-omics integration reveals that pyrimidine metabolism in lung adenocarcinoma drives an immunosuppressive microenvironment

Miaoyan Liu, Houqiang Li, Shenghan Xu, Yike Zhou, Min Yao, Jiahai Shi, and Lou Zhong

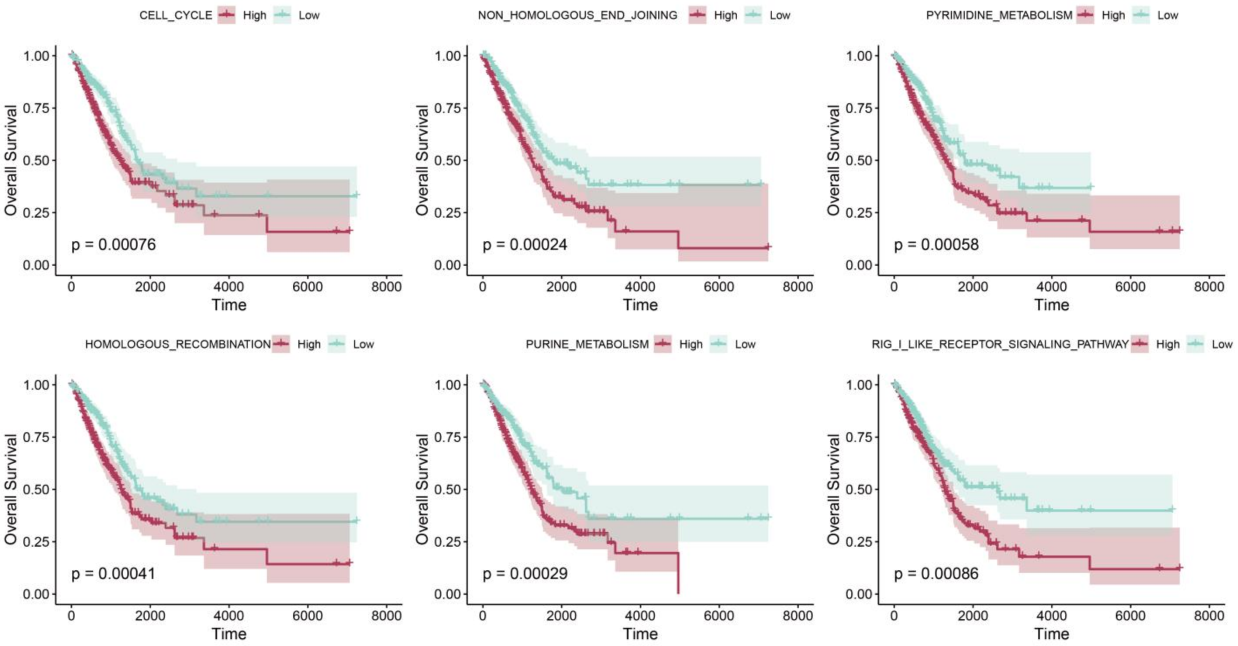

Figure S1. K-M curves for the scores of six metabolic pathways significantly associated with LUAD survival ($p < 0.001$).

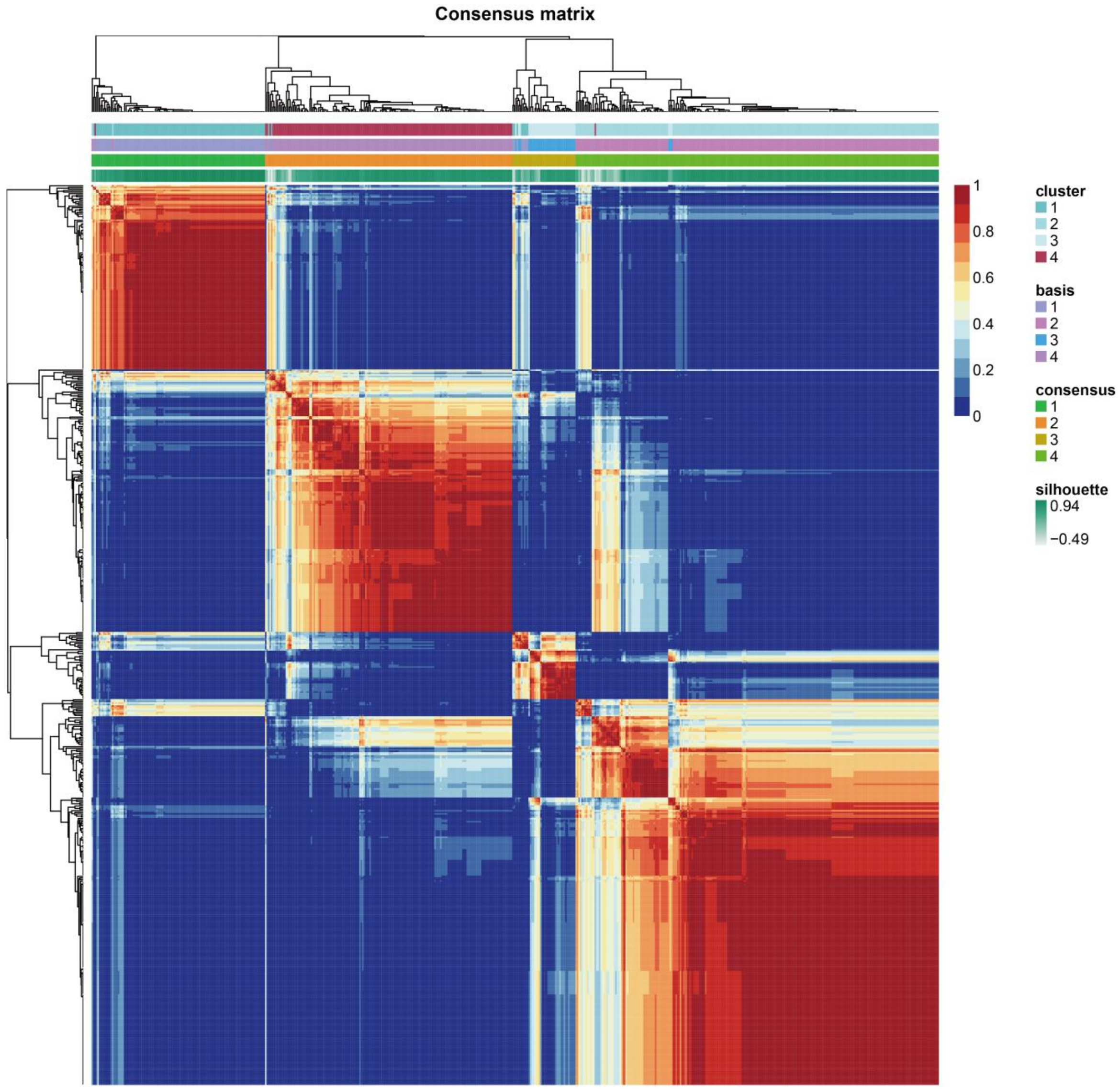

Figure S2. NMF (non-negative matrix factorization) clustering heat map.

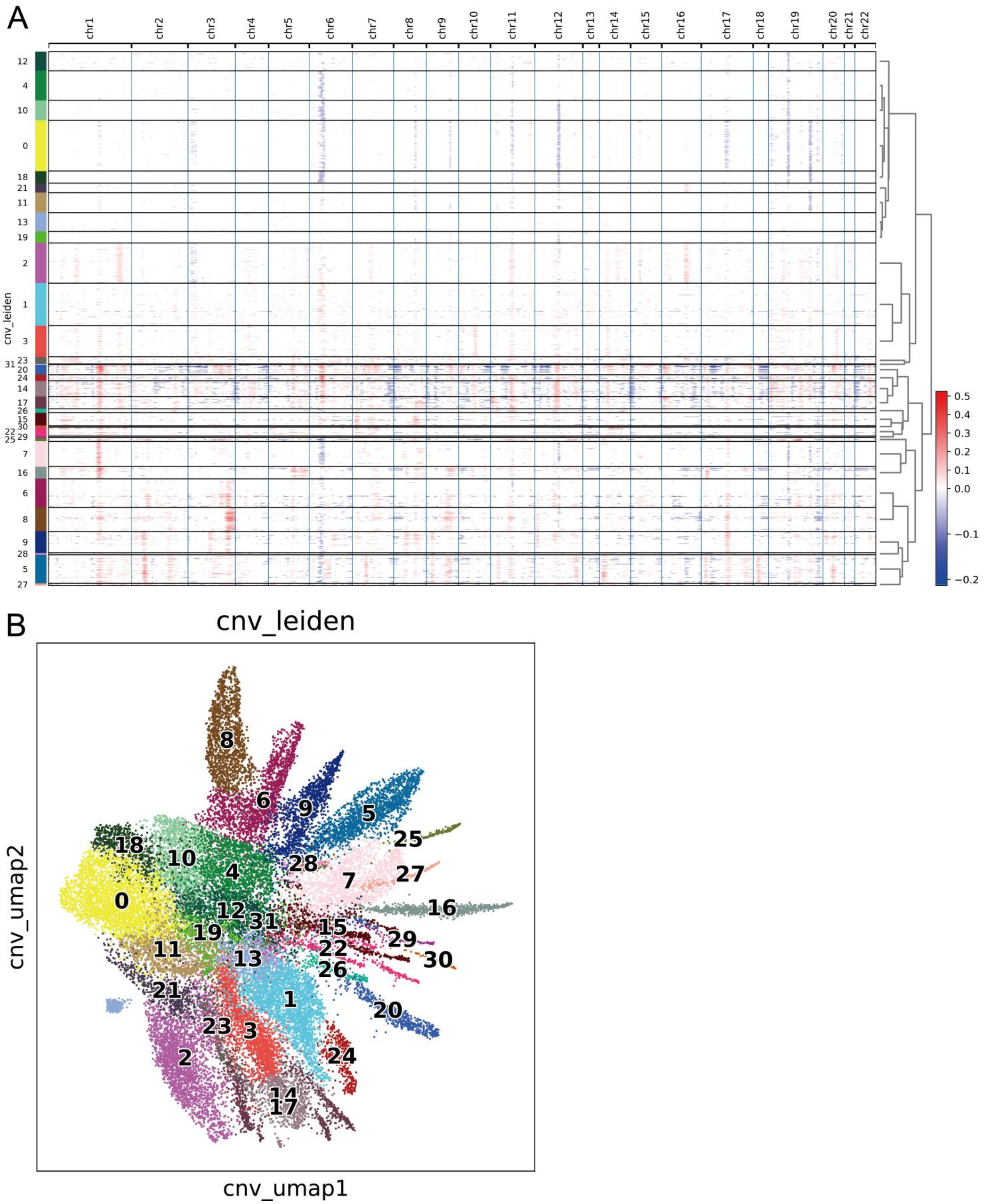

Figure S3. Copy number variation scores of LUAD epithelial cells. (A) LUAD epithelial cell copy number variation score clustering heat map. (B) LUAD epithelial cell copy number variation score Umap clustering diagram.

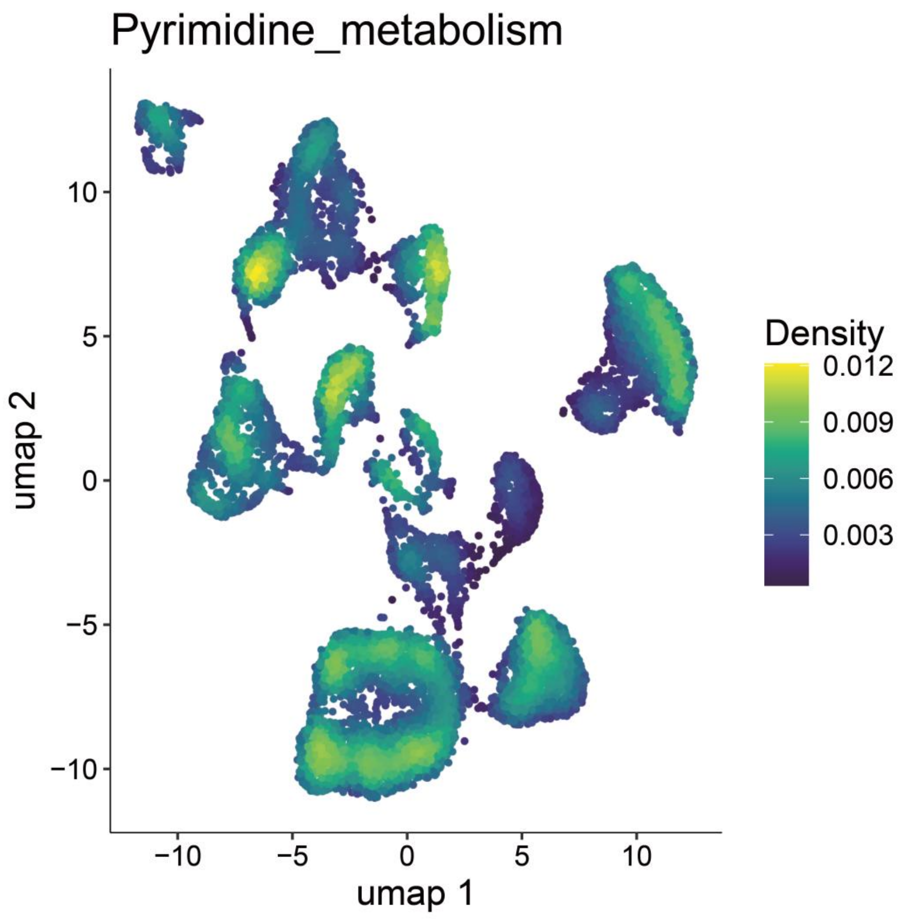

Figure S4. Pyrimidine metabolism score density map of malignant epithelial cells in LUAD

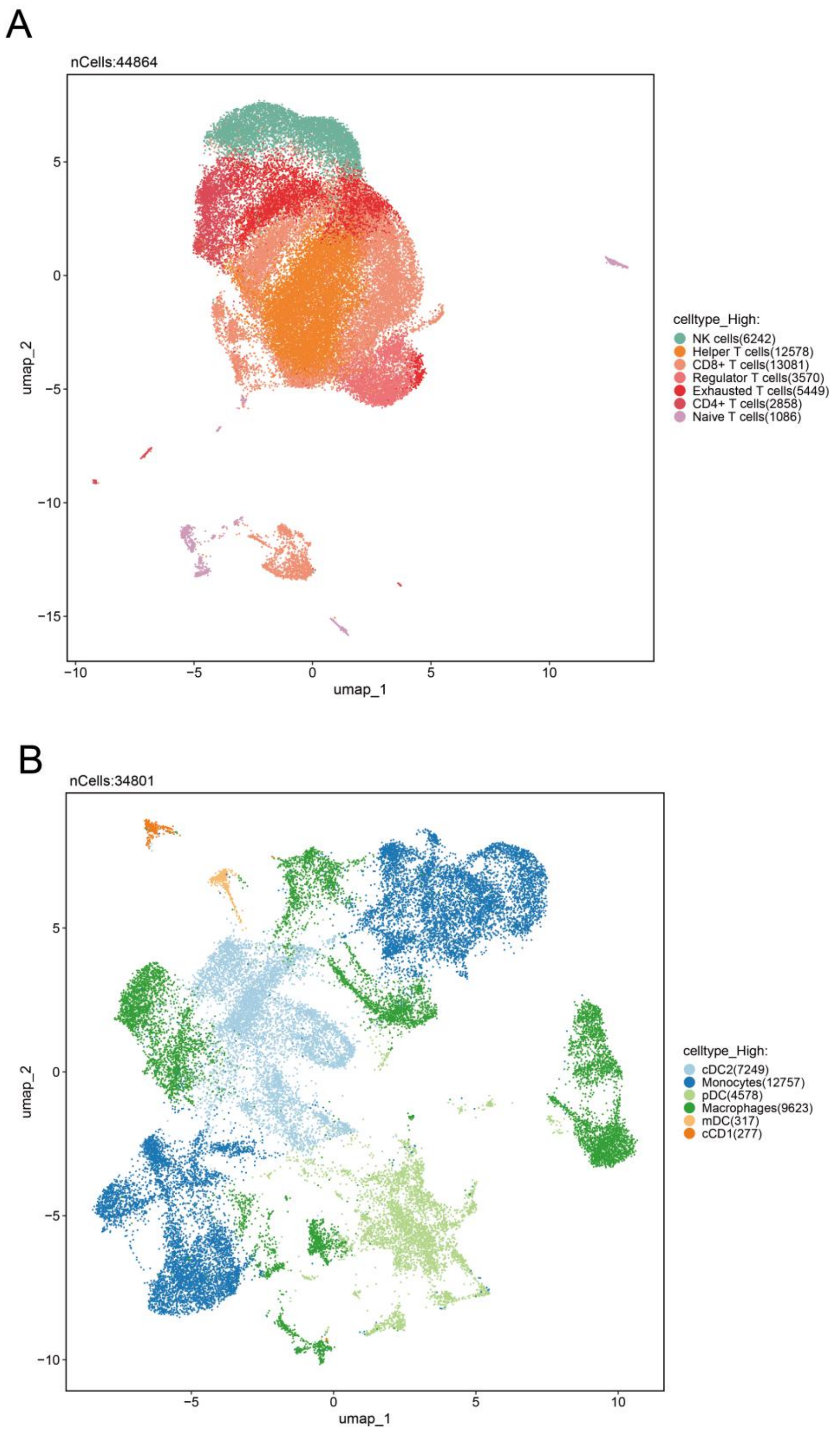

Figure S5. Immune cell subpopulations in LUAD patients visualized by UMAP. (A) UMAP plot of T/NK cell subpopulations in LUAD patients. (B) UMAP plot of myeloid cell subpopulations in LUAD patients.
